# The English Pseudoword Lexicon Project (EPLeP): A resource for investigating pseudoword processing

**DOI:** 10.3758/s13428-026-03151-5

**Published:** 2026-09-22

**Authors:** Fabio Marson, Rolando Bonandrini, Iva Šaban, Simona Amenta, Marco Marelli

**Affiliations:** 1 Department of Psychology, University of Milano-Bicocca, Piazza dell’Ateneo Nuovo 1, 20126 Milano, Italy; 2 NeuroMI, Milan Center for Neuroscience, Milano, Italy; 3 Department of Informatics, Systems and Communication – DISCo, University of Milano-Bicocca, Milano, Italy

**Keywords:** Lexical decision, English, Pseudowords, Megastudy, Morphology

## Abstract

In recent years, psycholinguistics has seen an increase in research focused on the processing of written stimuli that are not attested in the lexicon of a given language. We introduce the English Pseudoword Lexicon Project (EPLeP), a new database of lexical decision data for English pseudowords, nonwords, and words. While large-scale behavioral data have been collected for lexical and non-lexical items in English and other languages, none of these datasets have specifically focused on pseudowords. The EPLeP database fills this gap by considering three types of English-like non-lexical stimuli: (1) morphologically simple pseudowords generated from existing English words; (2) morphologically complex pseudowords obtained by combining existing stems and existing suffixes; and (3) nonwords, not following English graphotactics. This registered report provides an extensive overview of the steps adopted in producing non-lexical stimuli and the criteria for their selection. We collected data for almost 13,500 non-lexical and 4,400 lexical stimuli from 1,416 English-speaking participants across four online studies. Analyses of behavioral responses show that morphologically complex pseudowords trigger the deepest level of processing, whereas nonwords elicit the shallowest processing, reflecting a crucial difference in how these stimuli affect responses to words. This database will be a valuable tool for researchers interested in both pseudoword and word processing, enabling them to evaluate hypotheses by analyzing the collected behavioral data or using them to select stimuli for future studies. The Stage 1 protocol was accepted in principle on July 4, 2025 (https://osf.io/cz9rh/)

**Supplementary Information:**

The online version contains supplementary material available at https://doi.org/10.3758/s13428-026-03151-5.

## Introduction

The lexical decision task is a well-established tool in psycholinguistic research. During each trial, participants are presented with a sequence of letters and are instructed to determine whether it constitutes a real word or not (Meyer & Schvaneveldt, 1971). With regard to the identification of existing strings as “words”, the effect of different psycholinguistic features such as frequency, length, and orthographic neighborhood density on response times (RTs) and accuracy has been well documented and to this day remains undisputed (Balota et al., 2004; Keuleers et al., 2010; Murray & Forster, 2004; New et al., 2006). Conversely, lexical decision responses on nonwords or pseudowords (labels often used interchangeably in the literature) have received less attention.^1^ Two possible reasons could be suggested for the relative lack of interest in pseudoword/nonword recognition as opposed to words (Rastle et al., 2002).

First, words are among the most commonly studied types of stimuli in psychology, and they are widely recognized as units of meaningful content, indexing linguistic processing and yielding an enormous wealth of information (Balota et al., 2007; Balota et al., 2012; Rastle, 2016). As it happens, the very concept of “lexicon” refers (at least in its original description) to a “storage of known words,” i.e., a “mental dictionary” (for a review, see Coltheart et al., 2001; Morton, 1969; Treisman, 1960). Lexical decision has indeed been leveraged to describe the architecture of the mental lexicon by highlighting processing differences between words according to their phonological, orthographic, or semantic properties (see for instance Gardner et al., 1987; Kroll & Merves, 1986; Landauer & Freedman, 1968; Parkin, 1982; Rubenstein et al., 1970). Since—by definition—nonwords/pseudowords cannot be found in the lexicon (the fact that they do not elicit a sizable masked identity priming effect is typically taken as evidence for the absence of lexical activation; Forster, 1998) and have traditionally been conceptualized as meaningless, they have often simply served as foils in this task.

A possible second reason behind the comparatively smaller focus on pseudoword/nonword processing than on words is that the range of predictors that can be computed for pseudowords/nonwords is smaller than that for words. Indeed, pseudowords—just like words—can be characterized according to their length, their number of syllables, the size of their orthographic neighborhood, and the frequency of their subword units like bigrams or trigrams (see for instance Muncer et al., 2014; Yap et al., 2015), yet they have been traditionally associated with zero frequency of occurrence, no information of possible age of acquisition, and no semantic properties (but see Hendrix & Sun, 2021).

Still, the fact that pseudowords/nonwords are verbal stimuli that have never been encountered before and that, as such, they are not represented in the mental lexicon does not mean that they cannot be read at all, nor that they are unable to activate (at least to some extent) cognitive processes that are common to those involved in word reading. In this direction, it was shown that pseudowords/nonwords are not all alike: letter strings that do not follow the graphotactic rules of the target language (i.e., “nonwords”) make lexical decisions easier than non-lexical stimuli that obey graphotactic rules of said language (i.e., “pseudowords”; see Gibbs & Van Orden, 1998, Experiment 1). In a similar vein, it was shown that pseudowords comprising existing morphemes (e.g., *gasful*) are categorized as non-lexical stimuli more slowly than pseudowords that do not have a morphological structure (e.g., *gasfil*; Caramazza et al., 1988; Crepaldi et al., 2010; Taft & Forster, 1975). This shows that even nonexistent word-like stimuli may undergo decomposition into constituent morphemes, similarly to real words (e.g., “goodness”; “good” + “ness”). More recently, studies have specifically investigated lexical processing of pseudowords in the lexical decision task, suggesting overlooked complexities in how they are processed (Perea et al., 2005; Whaley, 1978; Yap et al., 2015). Analyses of response patterns for pseudowords/nonwords have mainly focused on three concepts: length, orthographic neighborhood density, and frequency of embedded elements (Yap et al., 2015). For instance, both longer pseudowords and pseudowords with a large number of real-word orthographic neighbors consistently result in longer response times in their classification as unattested strings of letters (Balota et al., 2004; Yap et al., 2015). Inhibitory effects of orthographic neighborhood density for nonwords have also been evidenced in the lexical decision task (Balota et al., 2004; Carreiras et al., 1997; Yap et al., 2015). Interestingly, it was shown that—just like words—pseudowords may give rise to frequency-like effects (Hendrix & Sun, 2021). In a similar vein, recent evidence has suggested that, although not present in the lexicon, pseudowords are not devoid of meaning. For instance, there is evidence for semantic effects in pseudoword processing, with slower response times for pseudowords with estimated denser semantic neighborhoods or more semantic loading (Bonandrini et al., 2023; Hendrix & Sun, 2021; Hsieh et al., 2024) and for pseudowords that activate semantic patterns that are related to a given prime word (Gatti et al., 2023).

This set of results suggests that pseudowords/nonwords are per se a potentially interesting object of study for psycholinguistics. More specifically, this wealth of recent data advocates for extensive psycholinguistic exploration of pseudoword processing while moving away from the traditional sublexical standpoint (Coltheart et al., 2001).

Such opportunity can be capitalized through the (re)analysis and/or modeling of pseudoword/nonword data from publicly available resources. In this regard, a number of studies in the literature have made available datasets that allow large-scale analysis of data for pseudowords/nonwords in a number of languages. Many of these works explored the processing of pseudowords by using them as target stimuli in megastudies (the name refers to the relatively high number of participants, items, or both; see e.g. Balota et al., 2012) to assess pronunciation of monosyllabic (Pritchard et al., 2012) and disyllabic (Mousikou et al., 2017) letter strings. However, these latter studies involved reading aloud, a task that—compared with lexical decision—is better suited for highlighting phonological (Katz et al., 2012) rather than lexical and semantic effects (Balota et al., 2004; Balota & Chumbley, 1984; Yap et al., 2012), especially in pseudoword processing (see also Ferrand et al., 2011; Mahé et al., 2015, 2018). Many megastudies^2^ employing lexical decision can also be found in the literature (see Table 1), which naturally leverage non-lexical stimuli to characterize the proposed task. Still, the focus of such megastudies is typically on the word stimuli; hence, they share some limitations which have a critical impact on the ability to explore visual pseudoword/nonword processing.

**Table 1:** Review of megastudies including visual pseudoword/nonword lexical decision data in different languages

Database Name	Reference	Language	*N*	#OPI (PW)	#W	#PNW	Pseudoword type(s)	Pseudoword/nonword generation strategy	Time limit (ms)	Feedback during task
No name given	Balota et al. 2004	English	60	>20	2,906	2,906	Monosyllabic pseudowords	Changing 1 to 3 letters in a corresponding word	No	After each trial
English Lexicon Project		English (US)	816	*M* = 27, *SD* = 6.91	40,481	40,481	Pseudowords: generated from words, which included morphologically simple and complex words	By changing one or two letters in a corresponding target word; the location of the letter change alternated across different words to include early, middle, and late positions	4,000	After each trial
British Lexicon Project	Keuleers et al. 2012	English (UK)	78	*M* = 41.29, *SD* = 9.31	28,730	27,137	Pseudowords from words, which could be morphologically complex	Wuggy	No	After each block
French Lexicon Project	Ferrand et al. 2010	French	975	*M* = 22.36, *SD* = 1.56	38,840	38,840	Pseudowords from words, which could be morphologically complex	From monosyllabic words: recombination of onsets and rhymes and extraction of items matching word stimuli according to orthographic and phonemic variables From polysyllabic words: recombination of syllables and extraction of items matching word stimuli	4,000	No
Chronolex	Ferrand et al. 2011	French	35	35	1,826	1,826	Pseudowords from monosyllabic monomorphemic words	Recombination of onsets and rhymes and extraction of items matching word stimuli according to orthographic and phonemic variables	No	No
MEGALEX	Ferrand et al. 2018	French	197	NA	28,466	28,466	Pseudowords from words, which could be morphologically complex	Lexique toolbox: randomization of bigrams or trigrams coming from real words	2,000	After each block
Dutch Lexicon Project	Keuleers et al. 2010	Dutch	39	39	14,089	14,089	Pseudowords from words, which could be morphologically complex	Wuggy	2,000	After each block
Dutch Lexicon Project 2	Brysbaert et al. 2016	Dutch	81	*M* = 40.5, *SD* =.5	30,016	29,601	Pseudowords from words, which could be morphologically complex	Wuggy	No	No
No name given	González-Nosti et al. 2014	Spanish	36	36	2,765	2,765	Pseudowords from words, which could be morphologically complex	Changing one letter of other words matched in frequency with those used in the study	2,000	No
Persian Lexicon Project	Nemati et al. 2022	Persian	113	NA	1,800	1,800	Pseudowords (no info on morphological complexity)	LINGUA software; creation of word-like pseudowords	No	NA
No name given	Soares et al. 2019	European Portuguese	55	55	1,920	1,920	Pseudowords (resemble EP words, no info on morphological complexity)	Changing letters in nonterminal position of the baseline word	2,500	No
Developmental Lexicon Project	Schröter and Schroeder, 2017	German	782	84 (adults only)	1,152 (for adults)	1,152	Pseudowords from words	Wuggy	No	No
Hebrew Lexicon Project	Stein et al., 2024	Hebrew	264	*M* = 35 *SD* = 2.5	10,000	10,000	Pseudowords from words	Shuffling 3 to *n*−1 letters of the baseline word	2,000	No
Malay Lexicon Project	Yap et al. 2010	Malay	44	NA	1,520 (for lexical decision task only)	1,520	Pseudowords from words	Replacing one letter from the baseline word	3,000	After each incorrect/time-out trial
Chinese Lexicon Project	Sze et al. 2014	Chinese (Mandarin)	35	*M* = 35	2,500 characters	2,500	Pseudo-characters from existing characters	Combining semantic components of existing characters	No	After each incorrect trial
Chinese Lexicon Project	Tse et al. 2017	Chinese (Cantonese)	594	*M* = 33	25,286 two-character compounds	25,085	Pseudo-characters from existing characters	Random recombination of characters	No	After each trial
No name given	Lee et al. 2015	Chinese (Mandarin)	180	*M* = 180	3,423	573	Nonexistent items created from radicals of real phonograms	Combining two radicals, or combining two radicals and changing one stroke	NA	NA

*N* = number of participants; #OPI = number of observations per item (pseudowords/nonwords); #W = number of word stimuli; #PNW = number of pseudoword/nonword stimuli; *M* = mean; *SD* = standard deviation; NA = not applicable

First, none of them has involved nonwords. Previous studies have shown differential activation of the visual word system when stimuli contain linguistic properties (e.g., Keuleers & Brysbaert, 2011; Ziegler et al., 1997), as seen with words and pseudowords, relative to stimuli that do not respect language graphotactics, such as nonwords. Second, although in a few studies pseudoword stimuli have been derived from word items, which could happen to be morphologically complex, morphological complexity has not been systematically controlled for pseudoword processing. Third, the lack of systematic control over the type of non-lexical stimuli could give rise to potential list effects (whereby a certain item is apparently processed better or worse just because it departs from all other stimuli in the list; see Keuleers & Brysbaert, 2011), potentially limiting the interpretability of data.

In order to overcome the main limitations of available resources and to foster the empirical exploration of pseudowords/nonwords processing, we conduct a lexical decision megastudy focused on different categories of pseudowords and nonwords. This registered report introduces the English Pseudoword Lexicon Project (EPLeP), offering a detailed overview of the generation of the stimuli, along with a plan for data collection of a behavioral database of lexical decision data for almost 13,500 pseudowords and an additional set of almost 4,400 words. The EPLeP aims at addressing the existing gap in the megastudy literature on lexical processing and providing data on visual recognition performance for three types of pseudowords:

1. Morphologically simple pseudowords generated from existing English words,
2. Morphologically complex pseudowords obtained by combining existing stems and existing suffixes, and
3. Nonwords that do not follow English graphotactics.

Critically, the proposed experimental design is focused on pseudoword/nonword processing and is aimed at keeping list effects under control for the categories of non-lexical stimuli of interest. The goal of the project is to produce a database accessible to researchers from diverse backgrounds (e.g., cognitive [neuro]science, psycholinguistics, computational modeling, linguistics) interested in the processing of novel letter strings. The structure of the experimental paradigm itself is designed to allow data to be used flexibly in both analyses of classical lexical decision performance and analyses of sequential priming (Buchanan et al., 2021). On the one hand, the availability of this resource will give researchers access to data through which they can evaluate (both statistically and computationally) new theoretical and/or applied frameworks dealing with how familiar and unfamiliar letter strings are processed. On the other hand, the resource will constitute a reference point for the development of future experimental and clinical tools: researchers/clinicians interested in constructing a task or a clinical test exploring pseudoword/nonword processing may use this database to select stimuli yielding a certain desired performance level.

## Materials and methods

### Participants

Participants will be native English speakers recruited via the Prolific platform. We expect to recruit a total of approximately 3,520 US-based participants. The inclusion criteria are as follows: age ≥ 18 and ≤ 50 years, having English as a first language, normal or corrected-to-normal vision, no language-related disorders, no diagnosis of dyslexia, and 90% Prolific approval rate. Experimental data will be complemented by sociodemographic data: Information about participants’ sex, ethnicity, nationality, country of birth, country of residence, and student/employment status will be retrieved from Prolific’s standard demographic information module. Information about participants’ age and education will be collected before the lexical decision task. Informed consent will be obtained from all participants included in the study. Only participants who successfully complete a practice section will be admitted to the main task (see below for further details). Participants will receive £2 for completing the study (estimated time 15 min). The ethical approval of the minimal-risk committee of the Department of Psychology, University of Milano-Bicocca, will be requested upon acceptance of this report, in order to adapt the request following potential amendments suggested by the reviewers.

### Stimuli

We devised a list of complex pseudowords (*N* = 4,499), a list of simple pseudowords (*N* = 4,524), a list of nonwords (*N* = 4,475), a list of simple words (*N* = 2,153), and a list of complex words (*N* = 2,225). Since we wanted to control for the comparability of lexical and non-lexical items according to a set of psycholinguistic features (i.e., length, orthographic neighborhood, frequency of embedded items [when available]), lexical items were extracted first, and the generation of non-lexical items ensued. This procedure also allows for large variability in terms of both psycholinguistic features and word-likeness, ensuring diverse application prospects in future studies. The information about the stimuli extraction procedures is outlined below, and more details can be found in the Supplementary materials.

#### Word stimuli

**Fig. 1:**
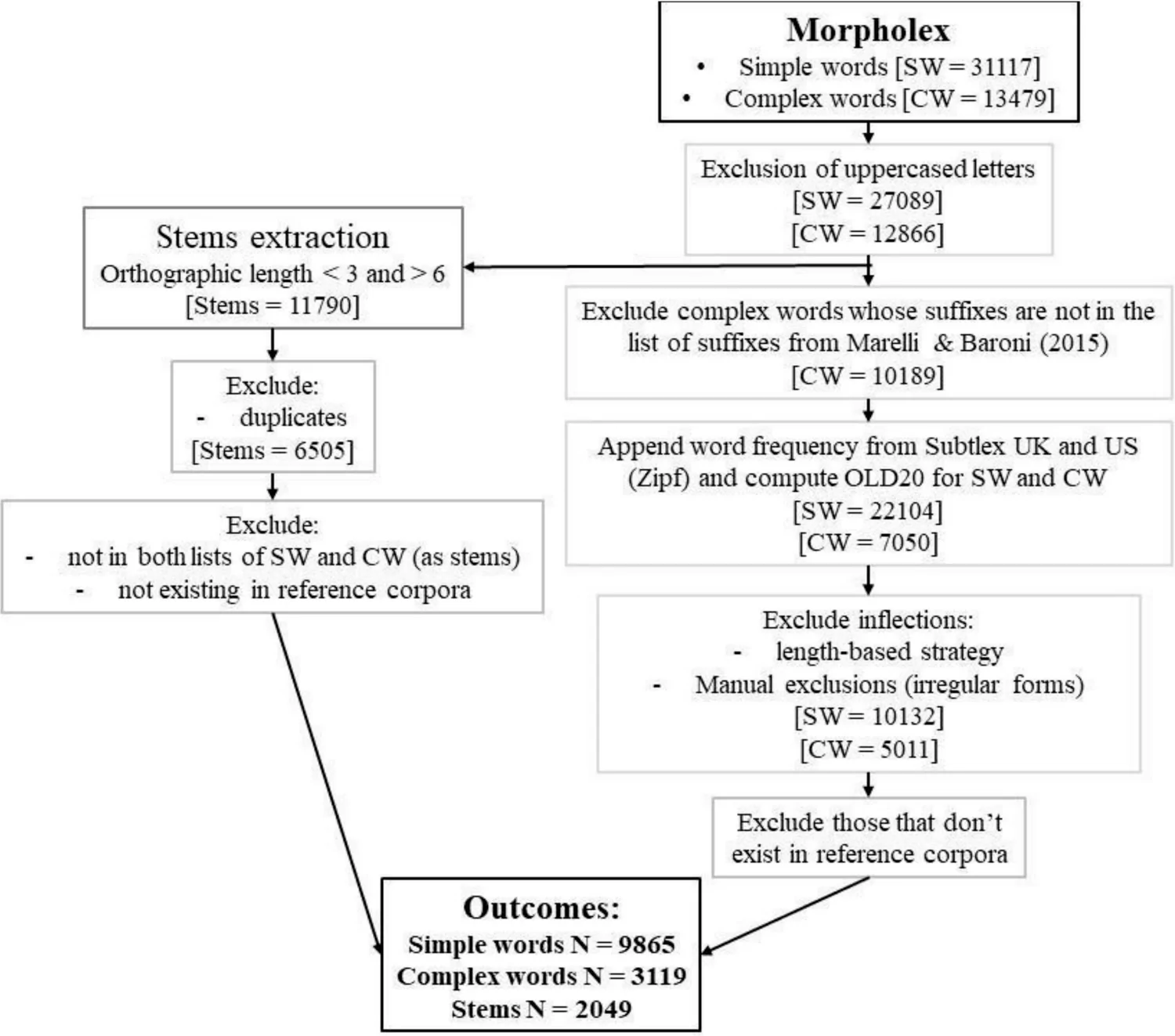
Pipeline for the preprocessing of stimuli detailing all of the exclusion criteria adopted from the extraction of stimuli from MorphoLex to three final lists of potential simple words, complex words, and stems. The number in square brackets is the number of words retained after each step

The first step in stimuli creation was the selection of words from the MorphoLex database (Sánchez-Gutiérrez et al., 2018), which contains annotations on the morphological complexity of its included items (i.e., number of prefixes, number of stems, number of affixes). We initially identified 31,117 non-affixed *simple words* (i.e., existing words that are not parsable into an existing stem + an affix, such as “age”) from the set of MorphoLex items with 0 prefixes, 1 stem, and 0 suffixes. In parallel, we also identified an initial set of 13,479 morphologically^3^*complex words* parsable in an existing stem and a suffix (i.e., “ageless” > “age” + “less”) from the set of MorphoLex items with 0 prefixes, 1 stem, and 1 suffix. In the selection of simple and complex words, we excluded all the items that contained uppercase letters (e.g., proper nouns), and items whose affix was not included in the list of common English suffixes considered in Marelli and Baroni (2015). In the next step, we excluded simple and complex words that did not appear in the SUBTLEX-UK (van Heuven et al., 2014) or SUBTLEX-US (Brysbaert et al., 2012) word frequency corpora. For the remaining words, we extracted the frequency in Zipf scale and computed the orthographic Levenshtein distance of the 20 nearest neighbors (OLD20), based on data from SUBTLEX-UK and SUBTLEX-US. We excluded inflected forms (e.g., plural forms, both regular and irregular, such as “dogs” or “men”; irregular verb tense form, such as “drove”). We made sure that all the words were represented in three reference corpora: the ukWaC (UK Web as Corpus; Baroni et al., 2009), the English Wikipedia-based corpus used to train fastText (see Bojanowski et al., 2017; available at https://fasttext.cc/docs/en/pretrained-vectors.html), and the Wuggy lexicon (Keuleers & Brysbaert, 2010). This procedure for word stimuli extraction is visualized in Fig. 1 (right panel). Only existing stems of the target length were extracted from the sets of simple and complex words, as visualized in the left panel of Fig. 1.

This intermediate stage resulted in a list of 9,865 morphologically simple words, a list of 3,119 morphologically complex words, and a list of 2,049 stems (the last for later use to produce complex pseudowords). These three sets of stimuli were eventually reduced through subsampling (for further detail, please see the *Word stimuli* section in the Supplementary materials) into a final list of 2,153 morphologically simple words, 2,225 morphologically complex words, and 200 stems.

#### Complex pseudowords

**Fig. 2:**
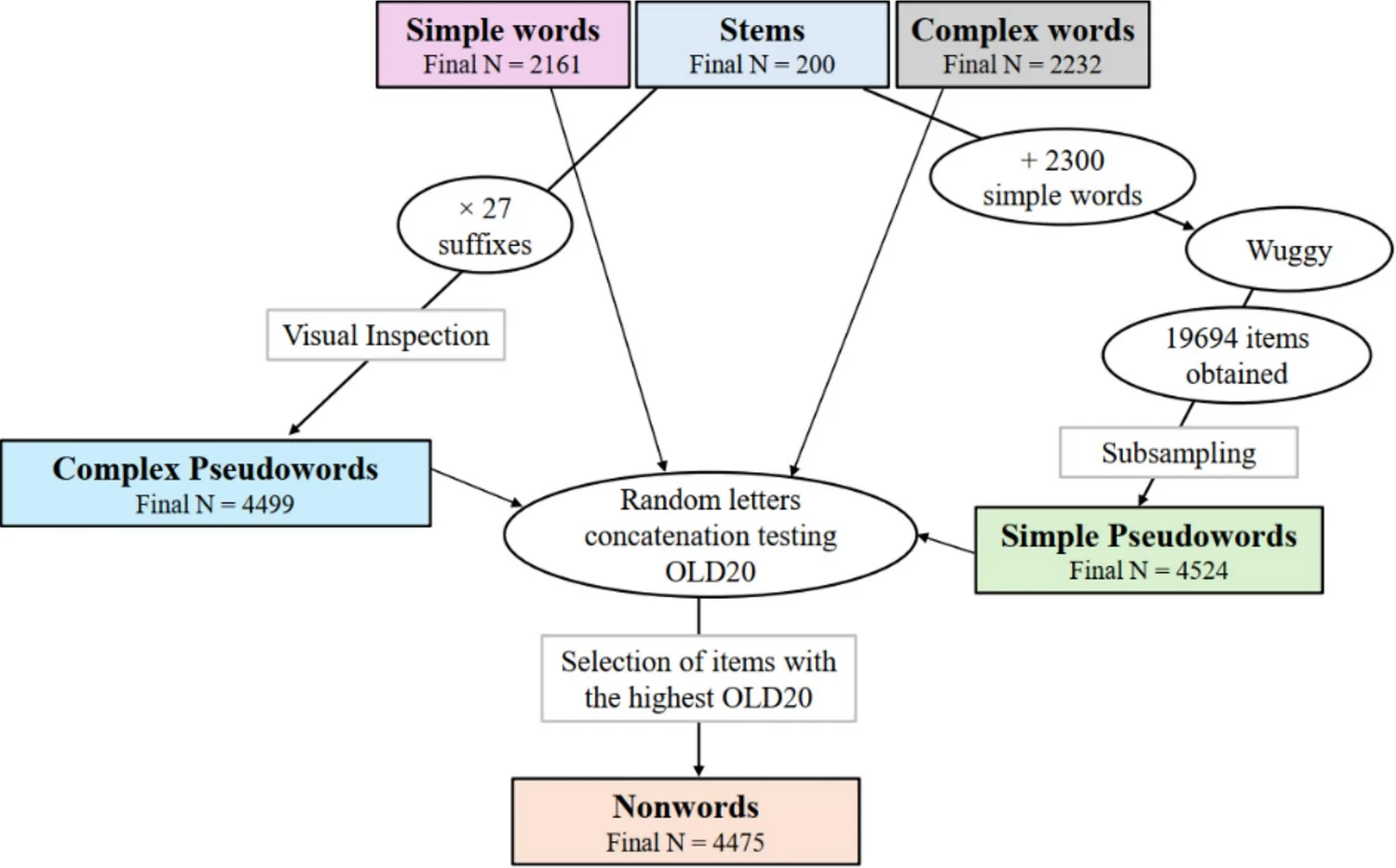
Outline of the processes adopted to create the lists of non-lexical stimuli

In the context of the present study, *complex pseudowords* are unattested letter strings that comply with the graphotactic rules of English and are created by appending existing stems to existing suffixes. To do so, we built on the list of 200 stems (see Fig. 2) extracted from MorphoLex. These stems ranged from three to six letters, so that, once combined with the suffixes, the length of the resulting complex pseudowords would not exceed 10 letters. By combining the stems with the 27 suffixes, we obtained a list of 5,400 complex pseudowords, for which we made sure that typical orthographic alterations in English affixation were respected (McCormick et al., 2008; see Table 2).

**Table 2:** Orthographic rules adopted for the creation of morphologically complex pseudowords, with corresponding examples

	Rule		Example		
Condition		Outcome	Stem	Affix	Final item
1	Stem's last syllable is stressed and has consonant-vowel-consonant structure (CVC)	Last consonant doubled	bag	-ery	baggery
2	Stem ending with “e” and suffix starting with vowel or “y”	“e” removed	life	-ify	lifify
3	Stem ending with a double consonant and suffix is -full/-less/-ly/-ness/-ment	Removed last letter of stem	puff	-ful	pufful
4	Stem ending with “y” and suffix is -ly	Ending “y” turns into “i”	fry	-ly	frily

We then checked that the resulting items were unattested in our reference corpora (i.e., MorphoLex, ukWaC, Wikipedia), and manually excluded unwanted inflected forms. This process yielded a final set of 4,499 complex pseudowords. On average, each stem was found in 22.52 complex pseudowords, and each suffix was repeated 166.85 times across this set of complex pseudowords.

#### Simple pseudowords

For the purposes of the present study, *simple pseudowords* are defined as unattested letter strings that comply with English graphotactic rules and for which no *existing stem* + *affix* structure can be identified. We generated our set of simple pseudowords using Wuggy^4^ (Keuleers & Brysbaert, 2010): 2,500 simple words were fed to Wuggy, which produced a total of 49,666 pseudowords. We then excluded pseudowords that were too long (> 10 letters), pseudowords appearing in the reference corpora, or pseudowords ending with one of the suffixes of interest or with an “s” (conceivable as an inflectional ending). This left us with 19,694 eligible simple pseudowords. After applying the subsampling procedure, we obtained a final list of 4,524 simple pseudowords, which matches our initially set target number of 4,500 simple pseudowords^5^ (for detailed information, see the *Simple pseudowords* section in the Supplementary materials). Three hundred of such simple pseudowords were also present in the British Lexicon Project (Keuleers et al., 2012), allowing for external validation of the stimuli (see Analyses section below).

#### Nonwords

This category of stimuli includes unattested strings of letters that do not follow English graphotactics. We sought to construct stimuli that were significantly less word-like than all other stimuli described so far by computing the OLD20 (i.e., Orthographic Levenshtein Distance 20, which represents the similarity between an item and the 20 most similar existing words; Yarkoni et al., 2008). We calculated OLD20 means and standard deviations for all four categories of stimuli and for each level of stimulus length (i.e., 3 to 10), where the lower the OLD20, the more word-like a stimulus is. For each level of stimulus length, we kept the highest OLD20 mean (and corresponding standard deviation) across the four different categories of stimuli as reference. In other words, for each length level, we considered the category of stimuli being the least word-like as the reference point to define nonwords. For each length level, we then generated 2,500 nonwords (see the section Nonwords in the Supplementary materials for more information) by randomly sampling letters (weighting for their frequency of occurrence in SUBTLEX-US) so that the resulting letter strings had a minimum OLD20 corresponding to the reference value for that length bin plus three standard deviations.^6^ For each length level, we selected the stimuli with the highest OLD20 values (i.e., the least word-looking items). We thus obtained a list of 4,475 nonwords.

### Megastudy design

The megastudy will be carried out in four separate data collection modules, corresponding to four separate experiments, as depicted in Table 3.

**Table 3:** The structure of four individual experiments with the corresponding number of trials

Study	Words (*N* = 4,378) *N*_*obs*_ = 175,120	Simple pseudowords	Complex pseudowords	Nonwords
Experiment 1	+	+ (*N* = 4,524) *N*_obs_ = 180,960	−	−
Experiment 2	+	−	+ (*N* = 4,499) *N*_obs_ = 179,960	−
Experiment 3	+	−	−	+ (*N* = 4,475) *N*_obs_ = 179,000
Experiment 4	+	+ (*N* = 1,500) *N*_obs_ = 60,000	+ (*N* = 1,500) *N*_obs_ = 60,000	+ (*N* = 1,500) *N*_obs_ = 60,000

Word stimuli are identical across the four experiments. The “+” sign indicates the presence of a given category of stimuli in the experimental set. The “−” sign indicates its absence. *N*_obs_ refers to the number of expected observations across experiments and pseudoword types

All four experiments will contain the same word stimuli. Conversely, the four experiments will vary in terms of non-lexical material: one will include morphologically simple pseudowords (Experiment 1; e.g., “plief,” “dokstine”), one will include morphologically complex pseudowords (Experiment 2; e.g., “plungable,” “snottant”), one will include nonwords (Experiment 3; e.g., “iprwvca,” “pghtmyg”), and one will include all three types of non-lexical items (Experiment 4). By adopting such study design, we maximize the number of non-lexical items, and at the same time we will be able to evaluate the effect of administering the same set of words when paired with different types of non-lexical stimuli. We note that illegal nonwords in Experiment 3 may lead participants to develop non-lexical decision strategies, based on their nonword-likeness (Gagl et al., 2022; Keuleers & Brysbaert, 2011). To quantify this effect, the same nonwords will be included in Experiment 4 alongside other non-lexical stimuli (i.e., simple and complex pseudowords) allowing for a between-experiment comparison (see the *Expected results* section).

In order to create the latter list with an equal number of items from each group of non-lexical stimuli, we subsampled all three categories (i.e., simple pseudowords, complex pseudowords, nonwords). This procedure produced a final set of 4,500 unattested stimuli for Experiment 4, composed of 1,500 simple pseudowords, 1,500 complex pseudowords, and 1,500 nonwords.

For each experiment, 22 experimental lists were compiled, each one containing 199 word stimuli (98 simple words, 101 complex words^7^) and approximately 206 non-lexical items (206 simple pseudowords or 204 complex pseudowords or 204 nonwords, for Experiments 1, 2, and 3, respectively). The construction of the experimental lists is described in more detail in the *Construction of experimental lists* section in the Supplementary materials).

Each experiment will be implemented and run online, using the PsyToolkit platform (Stoet, 2010, 2017). From each list, we defined the 10 most frequent words and the 10 non-lexical items with the highest OLD20 as catch trials, to ensure that the participants performed the task properly. For more information about the selection of catch trials, please see the *Catch trials* section in the Supplementary materials). Participants who fail to respond correctly to at least 7 out of 10 catch trials for both words and pseudowords will not be remunerated and will be excluded from subsequent analyses.

### Experimental design

The experimental task will be a lexical decision. Participants will be instructed to indicate whether a presented string of letters is a word or not, using American English spelling. The experiment will start with an intake session in which participants receive information about the experiment, give their consent to participate, and respond to a question about their highest education level. Participants will then be administered a practice session of 12 trials. For more information, please check the *Selection of practice trials* section in the Supplementary materials). The practice phase will allow participants to become familiar with the task and key assignment. Participants will be informed prior to the practice phase that if they do not achieve 100% accuracy in one of the practice cycles (12 trials), they will not be remunerated. As mentioned in the *Participants* section above, the practice phase will be terminated and no further data from this participant will be collected or analyzed if they do not reach 100% accuracy in a maximum of five attempts. During the practice phase (but not during the test phase), participants will receive feedback on their accuracy at the end of each trial.

**Fig. 3:**
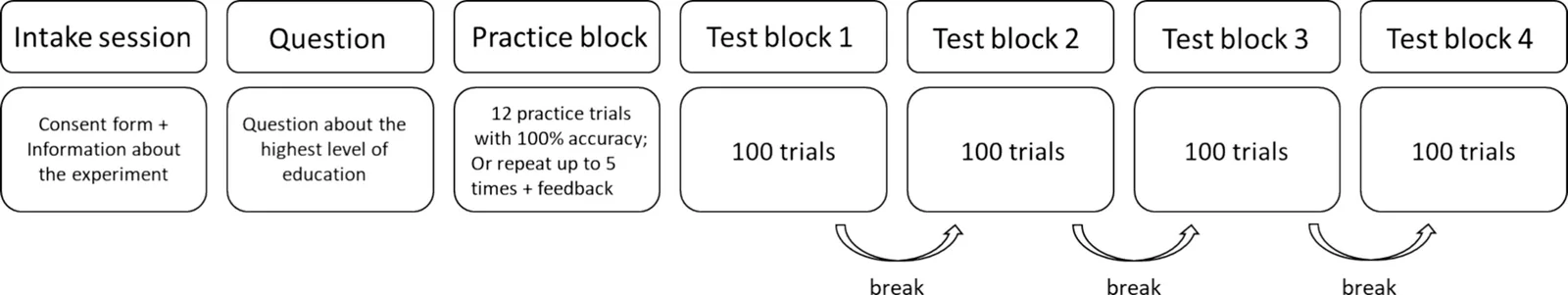
The structure of each individual experiment

The experiment will consist of five blocks (one practice and four test blocks), separated by short breaks every 100 trials. The first three test blocks will consist of 100 trials each (approximately 3 min; Keuleers et al., 2012). The number of trials in the fourth block may vary slightly to accommodate subtle differences in the number of stimuli between lists. The expected duration of the entire experiment is about 15 min. The structure of each experiment is presented in Fig. 3.

**Fig. 4:**
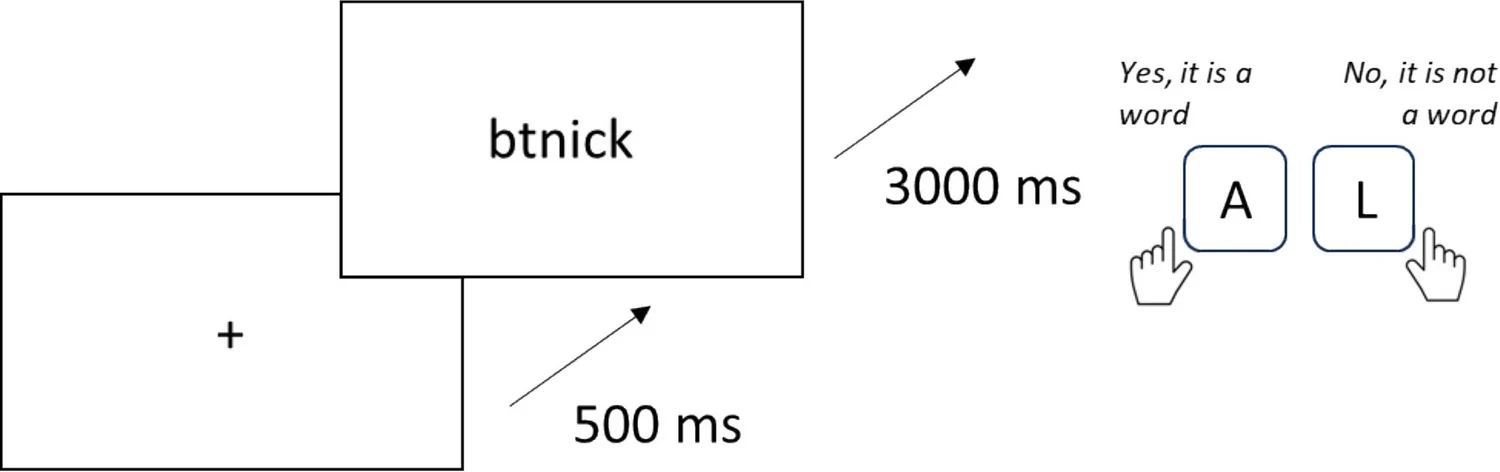
Structure of each experimental trial

Each trial will consist of the following sequence of events, visualized in Fig. 4. First, a fixation cross will appear in the center of the screen for 500 ms, after which the target stimulus will be presented until the participant makes a response or for a maximum of 3 s (Buchanan et al., 2021). Stimuli are presented in white on a gray screen in 50-pt Arial font. Participants will use the “A” key for “word” responses and the “L” key for “nonword” responses. For each specific stimulus list, the presentation order will be randomized across participants.

## Planned analyses and expected results

### Data collection and preprocessing

We plan to collect 40 observations per item (enough to ensure reliable data in a lexical decision task; see simulations in Amenta et al., 2025). Since we created 22 lists of stimuli and all presented items will be unique for each list, we expect to recruit 880 participants per experiment. Each participant will be allowed to complete a list only once, but the number of completions of other lists within the same experiment is not restricted. The total data (across four experiments) are expected to result in approximately 3,520 recruited participants, who provided responses to a total of 1,408,000 trials (704,000 word and pseudoword responses).

We expect that approximately 2–6% of trials (e.g., Keuleers et al., 2012; Mandera et al., 2020) will be eliminated from further analyses due to (1) technical issues or (2) being outliers. To ensure that extreme response latencies do not disproportionately influence the response times, we will exclude all response latencies faster than 200 ms and time-out responses. No time-out or missing values will be imputed during data collection. For evaluating the success/failure of catch trials (i.e., not for actual analyses), time-out responses will be coded as inaccurate. The number of excluded trials will be reported separately for each experiment. We will summarize statistics on the item level, such as type (pseudowords [simple, complex, or nonwords] vs. words [simple or complex]), length, accuracy, and raw RTs, based on the correct observations across the participants who received that particular item.

### Data analysis

#### Reliability and validity

These analyses are broadly intended to quantify the adequacy of the obtained dataset, both internally and with reference to external data.

We will determine the reliability of accuracy and RTs. Following previous megastudies (e.g., Ferrand et al., 2010, 2018), we will employ split-half correlation analysis. For each pseudoword, categorized by experiment, participants will be split into two groups (half A and half B), each comprising half of the participants exposed to that pseudoword. Subsequently, RT means and accuracies will be computed for each half. The item-level correlations will then be calculated between the two halves. Additionally, Spearman–Brown corrections will be applied and reported as *r*_corr_ = [2*r*/(1+*r*)]. Using this method, the reliability of the simple PW, complex PW, and nonwords will be reported separately for raw RTs and accuracy, along with their values after correction.

Data collected in the project will also be further validated by testing correlations of RTs for simple pseudowords with the RTs observed in the British Lexicon Project, including in the analysis those items shared between the two datasets (*N* = 300).

#### Lexicality analyses

These analyses will be performed to explore the effects of lexicality (i.e., lexical vs. non-lexical items) on both RTs and accuracy for the whole set of data. More specifically, we will include length and OLD20 as predictors and their interactions with lexicality. These analyses will be run both on by-item averaged data across participants (i.e., through linear models) and on item-level data (i.e., through linear mixed-effects models). This latter analysis will allow the exploration of potential practice effects on performance (i.e., change in accuracy/RTs throughout the testing session), both as a simple effect and as an interaction term with the other included variables.

#### Effect of pseudoword type on pseudoword processing

The goal of these analyses is twofold:

- Characterizing performance differences between different types of non-lexical stimuli, and
- Gauging potential effects of pseudoword type on the psycholinguistic variables affecting rejection of non-lexical stimuli.

They will be performed on RT and accuracy data for non-lexical stimuli only. We will include the variables length and OLD20, for which we will test the interactions with the variables pseudoword type (i.e., nonwords, simple pseudowords, complex pseudowords) and design (between-experiment [Experiments 1–3] vs. within-experiment [Experiment 4]). As for lexicality analyses, these analyses will be carried out both on by-item averaged data across participants (i.e., through linear models) and on item-level data (i.e., through linear mixed-effects models), to account for potential practice effects on performance, both as a simple effect and as an interaction term with the other included variables.

#### Effect of pseudoword type on word processing

The aims of these analyses are (a) to explore whether performance on words is affected by the type of non-lexical stimuli with which they appear and (b) to quantify potential effects of such pseudoword type on the psycholinguistic variables affecting lexical decisions on words. The analyses will be carried out on item-level (i.e., one data point per trial per participant) RT and accuracy data of lexical stimuli only, through linear mixed-effects models. We will include the variables length, OLD20, and frequency, for which we will test the interactions with the variable pseudoword type (i.e., the type of non-lexical stimuli [nonwords, simple pseudowords, complex pseudowords] with which word appeared in any given participant). Potential practice effects will be accounted for, both as a simple effect and as an interaction term with the other included variables. For these analyses we will focus on data from Experiments 1–3.

### Expected results

We assume that the use of orthographically illegal nonwords in Experiment 3 will result in a systematic difference between the lexical and non-lexical items, making them easily detectable (Gagl et al., 2022; Keuleers & Brysbaert, 2011). Experiment 3 will thus be used as a reference for comparison with Experiment 1 (including simple pseudowords) and Experiment 2 (including complex pseudowords). Consequently, we expect that participants will respond faster and more accurately to nonword items (Experiment 3) than to the pseudoword types used in Experiments 1 and 2. Apart from this cross-list comparison, we will further explore how lexical decision processes are related to orthographic legality by running Experiment 4, which includes a mix of all types of pseudowords adopted in the current study. In this final experiment, we expect to observe an enhancement of the effect of orthographic cues given the greater uncertainty in the experimental list (as all three types of non-lexical stimuli will be presented). Thus, in comparison with Experiment 3, we expect to observe a list effect that might provide insights about the role of orthographic cues in nonword processing while modulating the lexicosemantic context in which they appear.

We expect nonwords to be associated with the highest degree of reliability between participants. As for *validity*, we expect to find a significant positive correlation between performance in our study and the data from the British Lexicon Project.

Across all analyses, we expect to observe the effects of length and OLD20 (i.e., a facilitatory effect of length on words and an inhibitory one on pseudowords, and the opposite for OLD20; Hendrix & Sun, 2021) and practice effects (i.e., improvement in performance within the testing session; Keuleers et al., 2012). In addition, we expect to replicate the effect of frequency (i.e., facilitating performance; Brysbaert et al., 2011, 2018) on word stimuli (in the *effect of pseudoword type on word processing* analysis).

With regard to lexicality (*lexicality* analyses), we expect to observe a significant effect on both RTs (i.e., faster responses to words than nonwords) and accuracy (i.e., more errors for words than for nonwords; Keuleers et al., 2010; Stein et al., 2024). As for the effects of pseudoword type, in the *effect of pseudoword type on pseudoword processing* analyses, we expect to replicate previous findings suggesting that morphologically complex pseudowords are harder to reject than morphologically simple pseudowords (e.g., *gasfil*; Caramazza et al., 1988; Crepaldi et al., 2010; Taft & Forster, 1975). On the other hand, we expect nonwords to be associated with the highest level of performance across non-lexical stimuli. Indeed, it has been shown that patients with pure alexia (and who are therefore unable to read) have little or no difficulty in rejecting implausible nonwords in a lexical decision task (Ablinger & Domahs, 2009; Cohen & Dehaene, 2000).

In the *effect of pseudoword type on word processing* analyses, we expect pseudoword type to affect overall performance, with the degree of word-likeness (i.e., complex pseudowords, simple pseudowords, nonwords) of non-lexical stimuli to have a negative effect on the performance on words (see also Gibbs & Van Orden, 1998). In addition, we also expect pseudoword type to modulate the effects of the other psycholinguistic variables (i.e., length, OLD20, and frequency), particularly in line with previous evidence suggesting that the presence in the linguistic context of lexical vs. non-lexical stimuli affects the degree to which words are read through lexical vs. sublexical routes (Coltheart et al., 2001; Danelli et al., 2015). In this direction, we anticipate the length effect to be modulated across experiments in a complementary manner relative to that of the frequency effect. We assume that the frequency effect for words (diagnostic of lexical reading) will be more pronounced when words are presented among more word-like non-lexical stimuli (i.e., complex pseudowords, which would elicit a slower response than other non-lexical stimuli) than with less word-like non-lexical stimuli (i.e., nonwords, which conversely would elicit a faster response than other non-lexical stimuli). We also expect the length effect (diagnostic of sublexical reading) to be most evident whenever the least-word-like stimuli are used, and to decrease progressively in size as the word-likeliness of non-lexical stimuli increases. Since orthographic neighborhood effects are supposed to take place at an intermediate cognitive locus between visual analysis and lexical access (Ellis, 2004), we do not have a strong hypothesis on the nature of the possible interaction with pseudoword type. With regard to the effect of design (i.e., between- vs. within-experiment) in the *effect of pseudoword type on pseudowords* analyses, we do not have a priori hypotheses on the direction of the effect. These analyses therefore remain purely exploratory in nature.

As a final note, it is worth acknowledging that, unfortunately, prevalence data for pseudowords are not available. However, the present study provides an opportunity to estimate pseudo-prevalence for non-lexical items, based on their false recognition rates.

## Stage 2

### Deviations from Stage 1 registered report

Relative to the procedures outlined in the Stage 1 registered report, we note four deviations in Stage 2.

First, before data collection, we further excluded eight simple words and seven complex words from the item set; these were included by mistake since they failed the checks we devised for experimental stimuli (in particular, the taboo-ness and offensiveness checks, as outlined in Supplementary Materials). None of these words were used as stems for generating complex pseudowords, so their exclusion did not affect the set of non-lexical items. Moreover, after data collection, we noticed that two non-lexical items had been erroneously included as two different types of items. Specifically, the item “plumbery” was reported as complex pseudoword in Experiment 2 and as simple pseudoword in Experiment 1, while “friy” was reported as complex pseudoword in Experiment 2 and as simple pseudoword in Experiments 1 and 4. In the collected dataset, “plumbery” is reported only as a complex pseudoword and “friy” only as a simple pseudoword. This led to a slight change in the final number of collected data points across the four experiments, resulting in a total of 1,420,276 trials (700,461 word and 719,815 pseudoword responses).

Second, in the Stage 1 registered report, we stated that we would report a linear regression analysis on the effects of pseudoword types on pseudowords using by-item averaged data. However, since we cannot ensure independence of observations in by-item averaged data (e.g., pseudowords can appear in different experiments, and hence at different levels of the “design” predictor), we decided not to run such an analysis and only focus on (and report results of) the mixed-effects model.

Third, we included an ancillary, unplanned analysis of the *effect of pseudoword type on morphological effect in words*, since, following the planned analyses, it was deemed a potentially interesting further exploration of the data.

Finally, we note that we faced substantial convergence issues in the logistic mixed-effects models on accuracy. We attribute this to excessive model complexity (Bates et al., 2015) combined with a substantial lack of variance in data due to high overall accuracy (see below). For these reasons, for accuracy we report only the results of the linear model on by-item averaged data.

### Participants

We collected data from a total of 1,416 English speakers (55% women) based in the USA and recruited via the Prolific platform (mean age = 36.7 years, *SD* = 7.9 years, range 19–50).

### Data collection and preprocessing

**Fig. 5:**
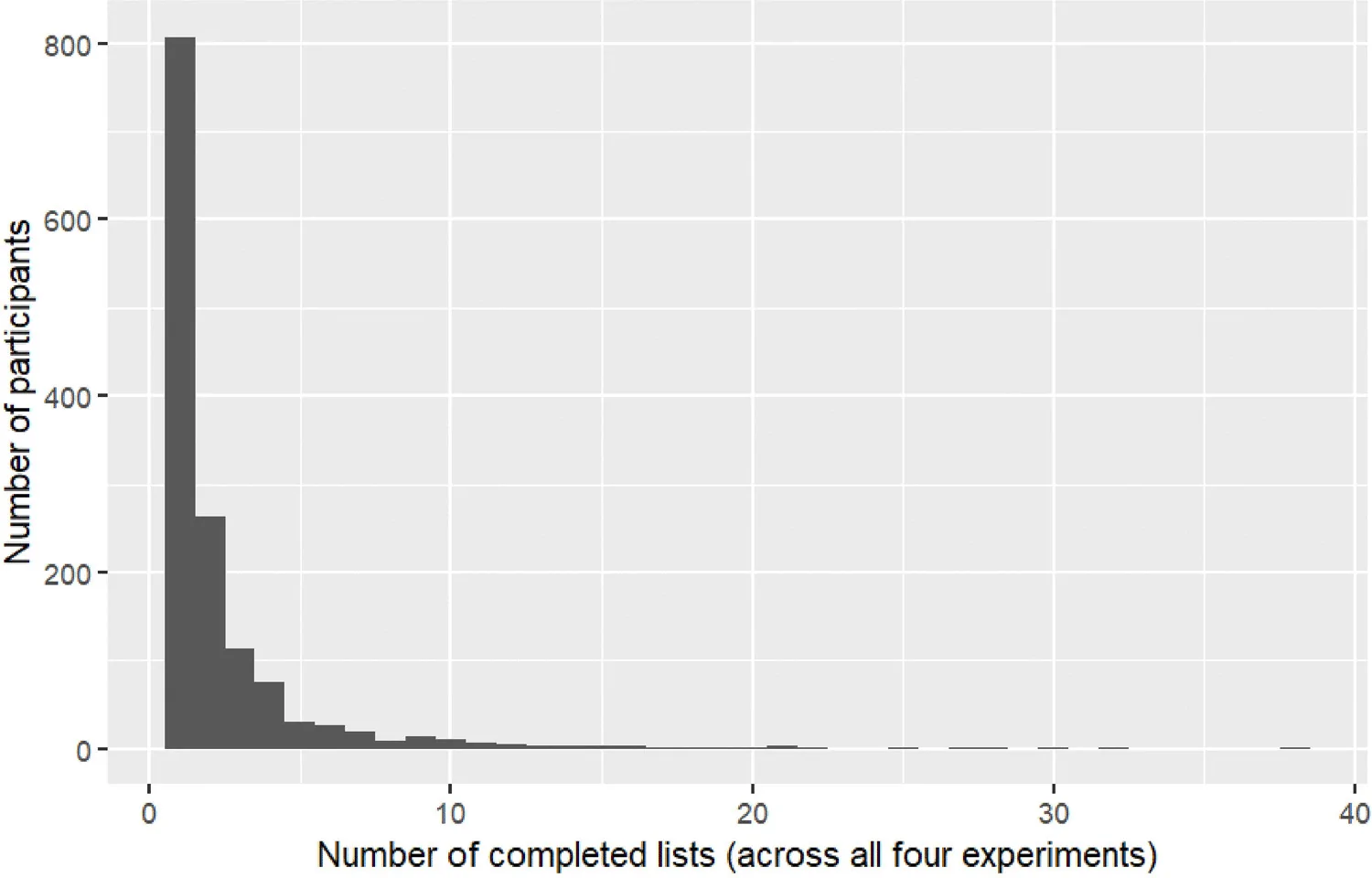
Distribution of the number of participants per completed experimental list across the four experiments

The ethical approval of the minimal-risk committee of the Department of Psychology, University of Milano-Bicocca was obtained prior to data collection (code RM-2025-1011). A total of 40 participants were recruited for each of the 88 unique experimental lists (across the four experiments). We thus collected a total of 3,520 completed tests. Participants took part, on average, in 2.48 (*SD* = 3.39) lists, with 807 unique participants who completed only one list and, at the other extreme, 13 participants who completed between 20 and 38 different lists. The distribution of the number of lists completed per participant is visualized in Fig. 5.

A total of 71 tests were discarded and were not analyzed, due to (i) failed practice session (*N* = 14), (ii) failed attention checks (*N* = 38), or (iii) technical issues (*N* = 19). This aligns with our estimation that approximately 2% of the collected data would have been eliminated from further analyses (e.g., Keuleers et al., 2012; Mandera et al., 2020).

**Fig. 6:**
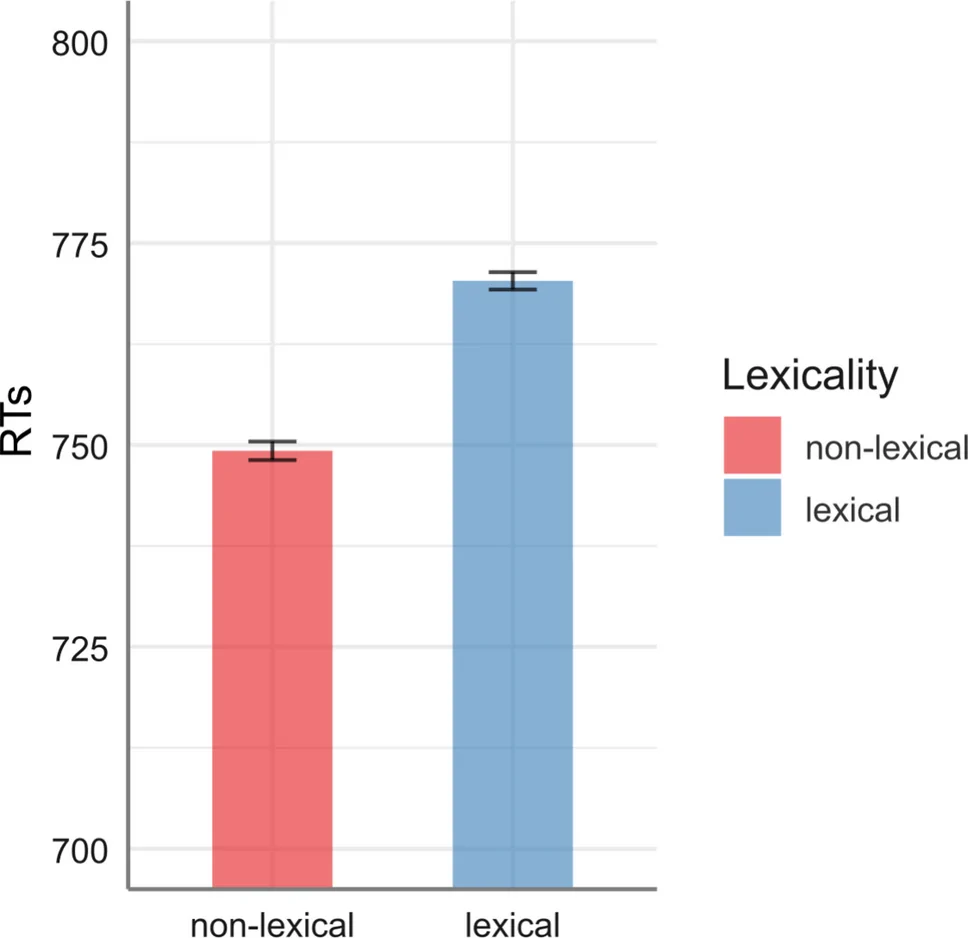
Mean RTs with standard errors for correct responses (non-lexical and lexical items)

**Fig. 7:**
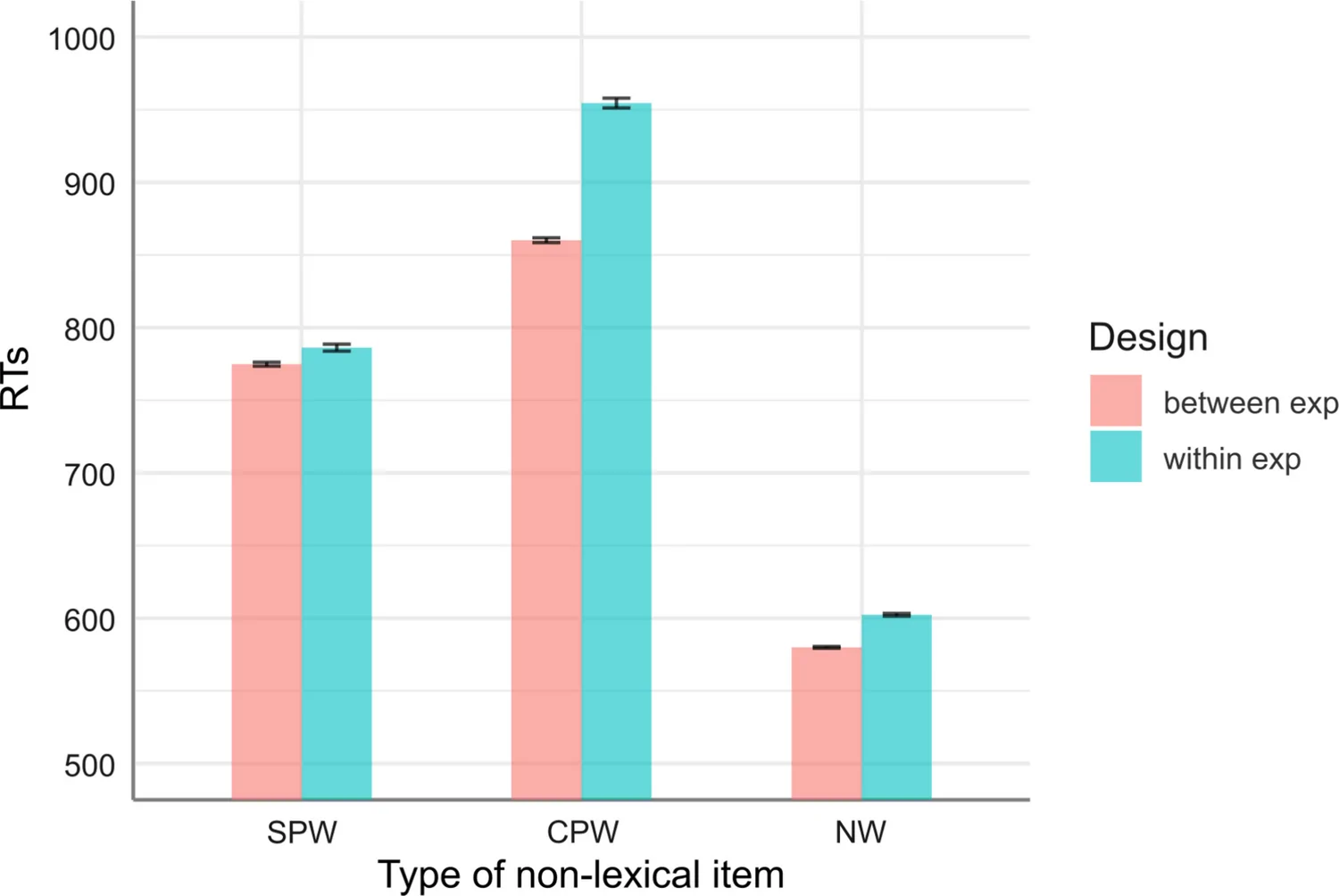
Mean RTs with standard errors for the different types of non-lexical items when presented as the only type of non-lexical items in the experiment (Experiments 1, 2, and 3: between experiment) and when all three types were presented in the same experiment (Experiment 4: within experiment). Abbreviations: SPW = simple pseudowords; CPW = complex pseudowords; NW = nonwords

**Fig. 8:**
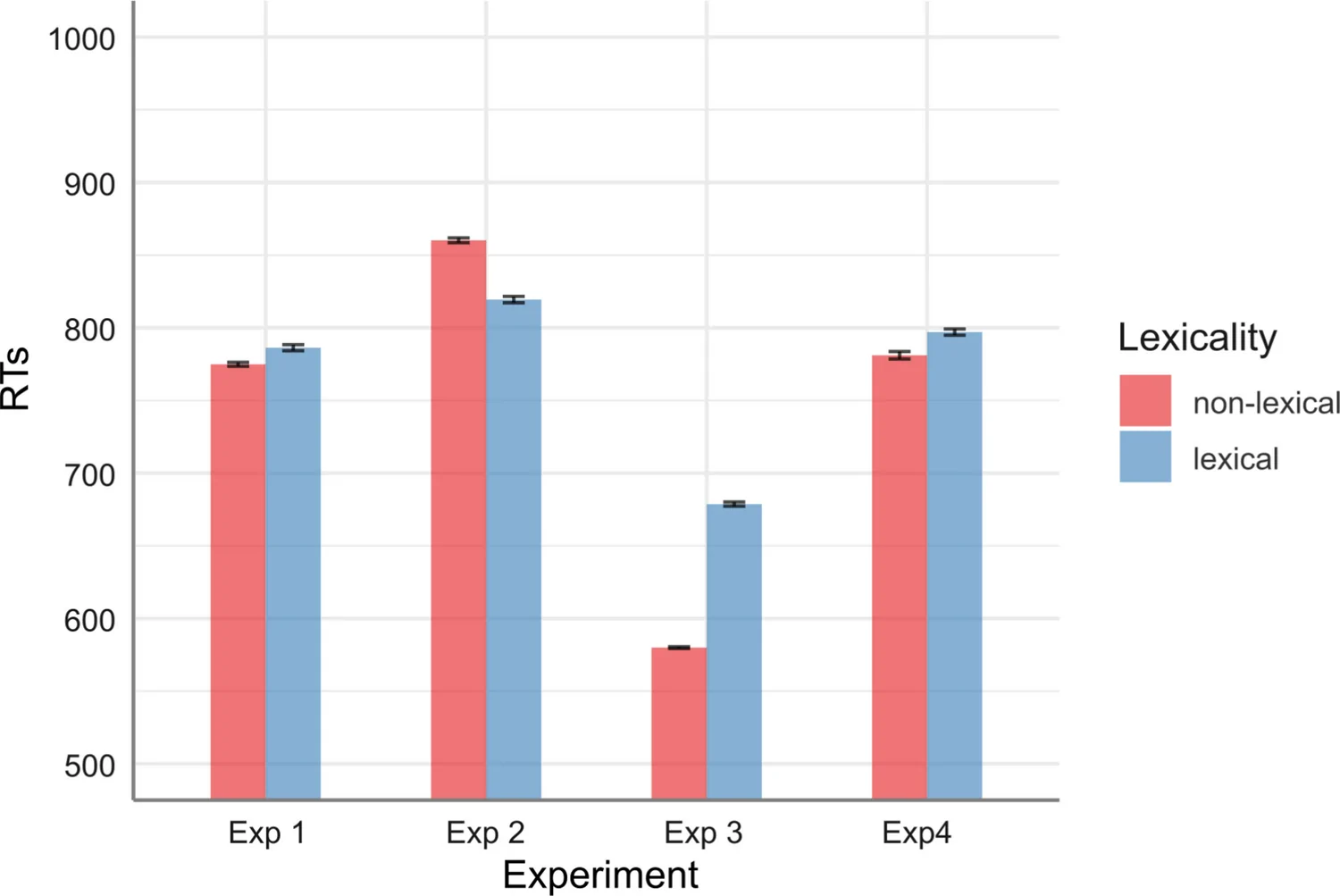
Mean RTs with standard errors for lexical and non-lexical items in four experiments. Non-lexical stimuli were simple pseudowords in Experiment 1, complex pseudowords in Experiment 2, and nonwords in Experiment 3, while in Experiment 4 they were a balanced combination of all three types of non-lexical stimuli (i.e., one-third simple pseudowords, one-third complex pseudowords, one-third nonwords)

We excluded all response latencies faster than 200 ms and time-out responses. The mean accuracy and response times for correct trials (both words and pseudowords) across the four experiments, together with the number of excluded trials, are reported in Table 4. Bar plots representing mean reaction times for correct responses and standard error for the stimuli included in our analyses in different experiments and conditions are reported in Figs. 6, 7, and 8.

**Table 4:** Trial-level descriptive statistics (mean accuracy and response times for correct responses, with standard deviation in brackets) after preprocessing

		Experiment 1	Experiment 2	Experiment 3	Experiment 4
Number of excluded non-lexical trials		721 (.40%)	777 (.43%)	432 (.24%)	809 (.45%)
Simple pseudowords	RT	775 (93)	–	–	786 (93)
	ACC	.96 (.06)	–	–	.96 (.06)
Complex pseudowords	RT	–	860 (111)	--	955 (131)
	ACC	–	.91 (.11)	--	.82 (.16)
Nonwords	RT	–	–	580 (39)	602 (36)
	ACC	–	–	.99 (.02)	.99 (.01)
Number of excluded lexical trials		664 (.38%)	615 (.35%)	488 (.28%)	773 (.44%)
Simple words	RT	791 (154)	797 (151)	683 (111)	787 (153)
	ACC	.81 (.24)	.78 (.26)	.90 (.15)	.83 (.23)
Complex words	RT	781 (120)	841 (136)	674 (77)	806 (131)
	ACC	.89 (.16)	.81 (.21)	.94 (.08)	.88 (.17)

RT = response times, ACC = accuracy, empty cells marked with “–” indicate that type of pseudoword was not used

### Results

#### Reliability

For each experiment separately, we computed the split-half reliability for RTs and accuracy for each pseudoword. Participants were split into two groups—one comprising the first half of those who saw a given item, and other comprising the remaining half—following Ferrand et al. (2010). Subsequently, average RT and accuracy were computed for each half, and the item-level correlations were calculated between the two halves. Additionally, Spearman–Brown corrections were applied and reported as *r*corr = [2*r*/(1+*r*)]. The reliability of the set from each experiment is reported separately for raw RTs and accuracy, along with their values after correction (see Table 5).

**Table 5:** Reliability indices for response times (correct responses only) and accuracy for each pseudoword type across experiments

Pseudoword type	Experiment 1		Experiment 2		Experiment 3		Experiment 4					
	Simple		Complex		Nonword		Simple		Complex		Nonword	
	RT	ACC	RT	ACC	RT	ACC	RT	ACC	RT	ACC	RT	ACC
*r*(raw)	.63	.63	.65	.77	.17	.009	.62	.59	.59	.79	.23	.01
rcorr	.77	.78	.79	.87	.29	.018	.76	.74	.75	.88	.38	.02
Number of unique PWs	4523		4498		4475		1500		1500		1500	

*r*(raw) represents correlations between half A and half B; rcorr indicates the reliability index after Spearman–Brown correction

As reported in the table, the reliability of response times and accuracy for simple and complex pseudowords (in Experiments 1 and 2, respectively) is comparable to those for words reported by Ferrand et al. (2010, 2018). However, reliability for nonwords (e.g., Experiment 3) was substantially lower (*r* ≈.20 for RTs and *r* ≈.01 for accuracy), which is likely due to ceiling effects in performance (Schröter & Schroeder, 2017). In RT data, the ceiling effect is reflected in reduced variability, which is consistent with the lower SD observed for the non-lexical items in Experiment 3 relative to the other experiments (e.g., see Table 4). Also, low reliability values for nonwords (see Table 5) differ from our expectations and indicate that most of the variance we observe for reaction times and accuracy in processing nonwords amounts to noise. The pattern of reliability for different stimulus types is largely consistent between the lists in which only one type of non-lexical stimuli is used (i.e., Experiment 1–3) and when these are presented in the same list (i.e., Experiment 4).

#### Validity

For the sake of external validation, we tested correlations of RTs and accuracy between pseudowords from the present work and corresponding stimuli from the British Lexicon Project (BLP; Keuleers et al., 2012). We first checked these correlations considering a set of 300 simple pseudowords appearing in both the BLP and our Experiment 1. Significant correlations were found for both RTs (*r* =.59, *p* <.001) and accuracy (*r* =.69, *p* <.001). We also observed significant correlations in both RTs (*r* =.71, *p* <.001) and accuracy (*r* =.74, *p* <.001) for a smaller set of 107 simple pseudowords that appeared in both our Experiment 4 and the BLP. The obtained results thus confirmed our predictions of significant positive correlations between pseudoword performance in the present series of experiments and the BLP with regard to the validity of the collected data.

To provide a benchmark for interpretation, we computed correlations for 1,292 nonwords shared between the English Lexicon Project (ELP) and the BLP. This revealed significant correlations for both raw response times (*r* =.54, *p* <.001) and accuracy (*r* =.72, *p* <.001), which are comparable to those observed in the present dataset and consistent with good external validity.

#### Lexicality analyses

Data were analyzed in the R environment (https://www.r-project.org/). The full datasets and analysis scripts are available at the OSF page of the project (https://osf.io/cz9rh/) . The analyses reported below were conducted using the lme4 package in R (Bates et al., 2015). In all analyses, the factor gauging stimulus type was dummy (treatment)-coded (the level “pseudoword” served as baseline in *lexicality* analyses, the level “simple pseudowords” served as baseline in the *effect of pseudoword type on pseudoword processing* analyses, and the level “complex pseudowords” served as baseline in the *effect of pseudoword type on word processing* analyses*,* whereas Experiment 1 served as baseline in the *effect of pseudoword type on morphological effect in words* analyses). Importantly, in all analyses, *F* values and related significance were interpreted. The plots for the present and subsequent analyses were obtained from estimated values derived from regression parameters.

**Fig. 9:**
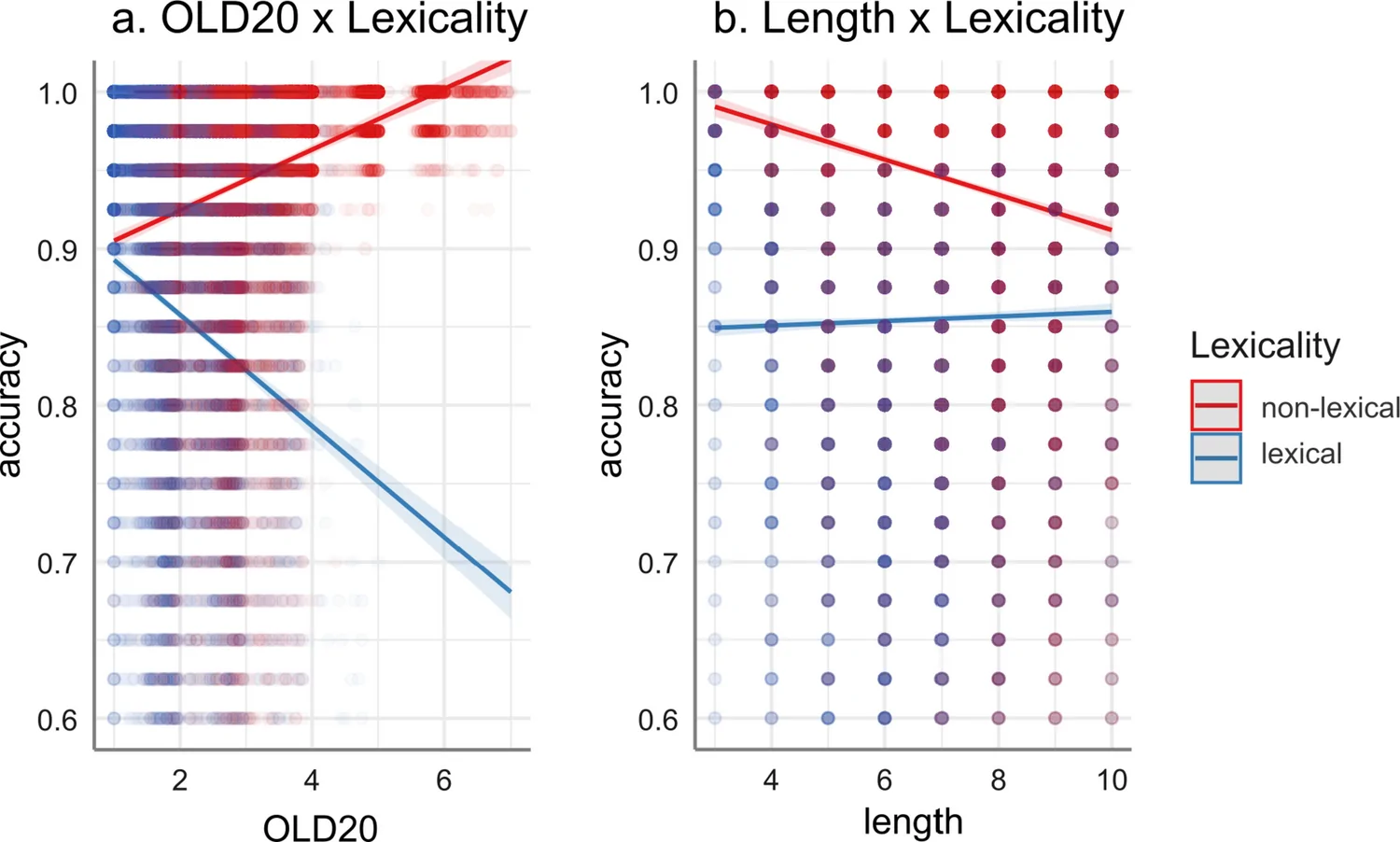
Results for accuracy in the analyses on by-item averaged data showing the interaction of lexicality × OLD20 (panel **a**) and lexicality × length (panel **b**). Dots represent data points averaged by item

**Fig. 10:**
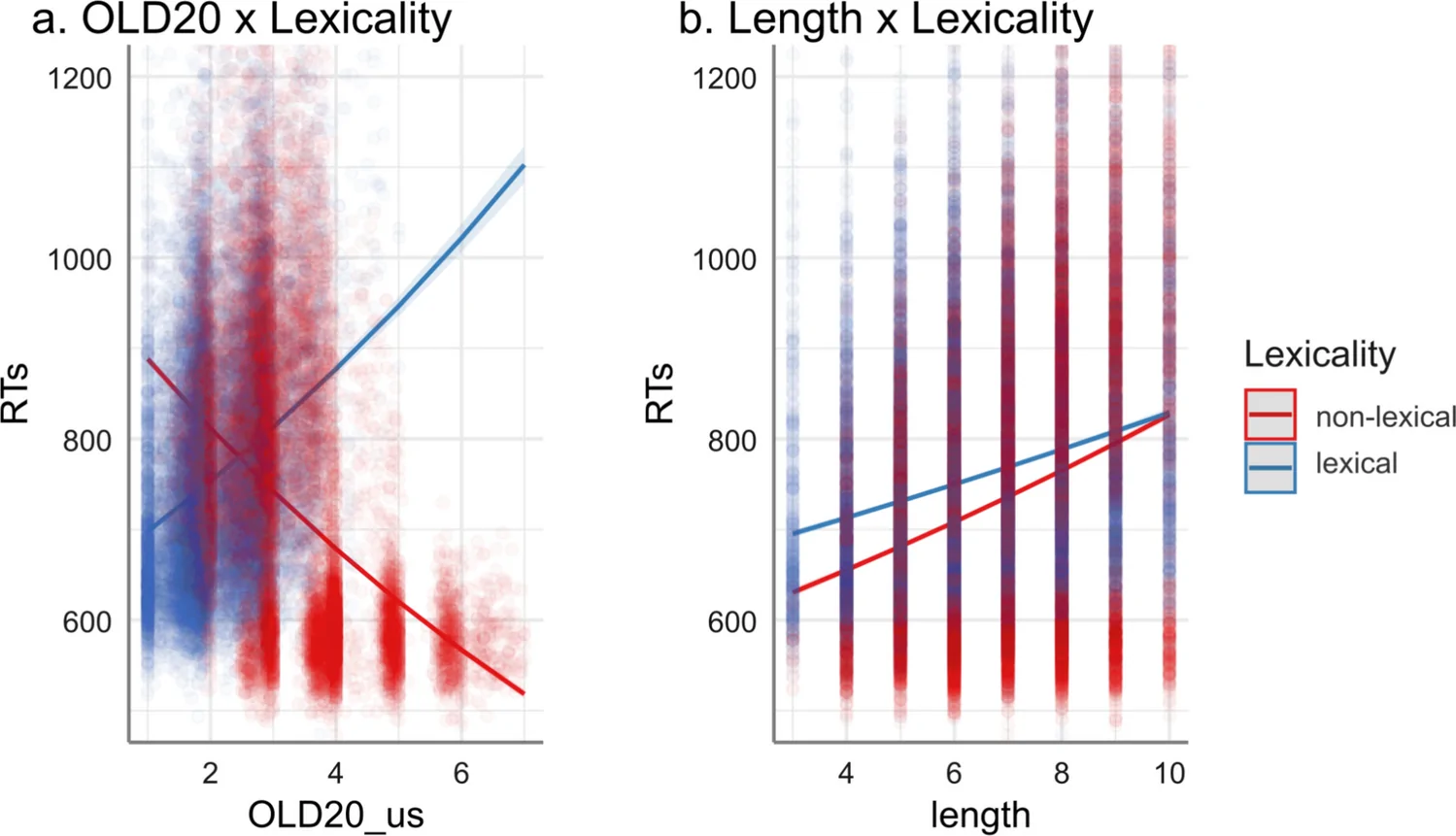
Results for reaction times in the analyses by-item averaged data showing the interaction of lexicality × OLD20 (panel **a**) and lexicality × length (panel **b**). Dots represent data points averaged by item

Here we report the results of a series of models conducted on the full dataset, contrasting 4,378 lexical and 13,496 non-lexical items. We first report linear model analyses on by-item averaged data across participants for response times and accuracy. To avoid collinearity issues, we included OLD20 (Table 6) and length (Table 7) as predictors in separate models, together with their interactions with lexicality. These results are also visualized in Figs. 9 and 10.

**Table 6:** Linear models with OLD20 as predictor for response times and accuracy

	Response times					Accuracy				
	*Df*	Sum Sq	Mean Sq	*F* value	Pr(>*F*)	*Df*	Sum Sq	Mean Sq	*F* value	Pr(>*F*)
Lexicality	1	9.59	9.59	330.32	<.001	1	75.35	75.35	3104.21	<.001
OLD20_us	1	53.25	53.25	1834.72	<.001	1	0.46	0.46	18.94	<.001
Lexicality × OLD20_us	1	157.28	157.28	5419.48	<.001	1	17.12	17.12	705.17	<.001

**Table 7:** Linear models with length as predictor for response times and accuracy

	Response times					Accuracy				
	*Df*	Sum Sq	Mean Sq	*F* value	Pr(>F)	*Df*	Sum Sq	Mean Sq	*F* value	Pr(>F)
Lexicality	1	9.59	9.59	296.18	<.001	1	75.35	75.35	3060.19	<.001
Length	1	87.69	87.69	2709.09	<.001	1	1.61	1.61	65.35	<.001
Lexicality × length	1	4.10	4.10	126.54	<.001	1	3.57	3.57	145.08	<.001

We assessed the lexicality effect, a typically highly replicable effect in visual word recognition, according to which lexical items should elicit faster and more accurate responses than non-lexical items (Forster & Chambers, 1973; Monsell et al., 1989). Contrary to our expectations, we observed a reversed lexicality effect on RTs, with non-lexical items being responded to faster than lexical items (see also Fig. 6). This could be explained by the presence of nonwords, orthographically illegal items on which participants performed at ceiling (i.e., extremely fast rejection time with almost no mistakes), which “drag down” the response latencies for non-lexical items (reducing the average RT), and/or to response strategies enacted by participants to best adapt to the specific task and list composition.

As expected, we observed the effects of OLD20 and length on both RTs and accuracy. Responses to non-lexical items with lower OLD20 were less accurate (Fig. 9, panel a) and slower (Fig. 10, panel a), with the opposite effect for lexical items. Responses to longer items (both words and pseudowords) were slower (Fig. 10, panel b) and less accurate, notably for non-lexical items (Fig. 9, panel b).

**Fig. 11:**
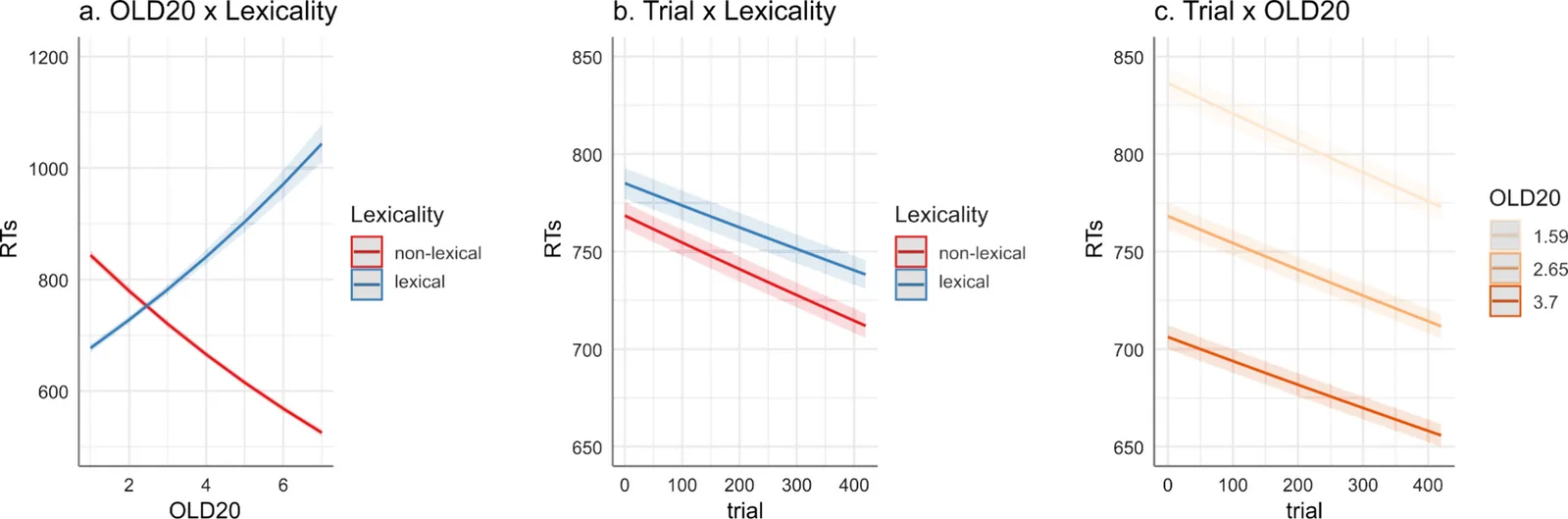
Plot of significant interactions from the OLD20 model reporting OLD20 × lexicality (**a**), trial × lexicality (**b**), and trial × OLD20 (**c**)

**Fig. 12:**
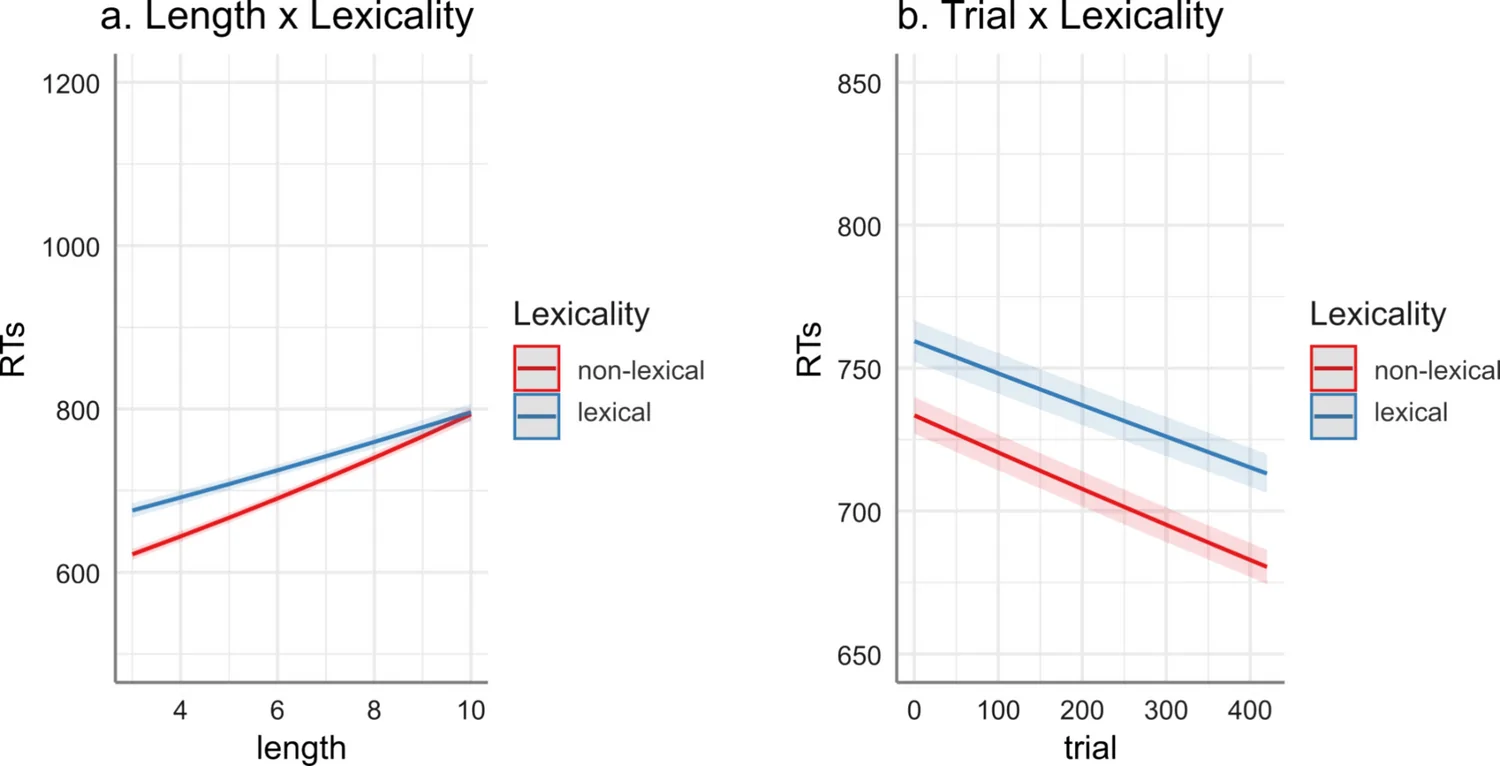
Plot of significant interactions from the length model reporting length × lexicality (**a**) and trial × lexicality (**b**)

We then report response time analyses on item-level data through linear mixed-effects models. As in the previous analysis, we assessed the effect of lexicality on one of the psycholinguistic variables (either OLD20 or length) along with their interaction (see Figs. 11 and 12 and Tables 8 and 9, respectively) in separate models.

**Table 8:** Results for the linear mixed-effects model on reaction times including OLD20 as predictor

	Sum Sq	Mean Sq	NumDF	DenDF	*F* value	Pr(>*F*)
Lexicality	141.47	141.47	1	17107	2245.63	<.001
OLD20_us	0.42	0.42	1	18712	6.72	.009
Trial	58.04	58.04	1	1261594	921.37	<.001
Lexicality × OLD20_us	127.08	127.08	1	16511	2017.25	<.001
Lexicality × trial	4.00	4.00	1	1261209	63.49	<.001
OLD20_us × trial	0.38	0.38	1	1261617	6.03	0.014

**Table 9:** Results for the linear mixed-effects model on reaction times including length as predictor

	Sum Sq	Mean Sq	NumDF	DenDF	*F* value	Pr(>*F*)
Lexicality	6.25	6.25	1	16991	99.15	<.001
Length	75.68	75.68	1	19914	1201.31	<.001
Trial	20.10	20.10	1	1260651	319.13	<.001
Lexicality × length	3.06	3.06	1	16792	48.54	<.001
Lexicality × trial	3.47	3.47	1	1260641	55.14	<.001
Length × trial	0.24	0.24	1	1260688	3.78	.052

These models are particularly useful in capturing effects that unfold over the course of an experiment (e.g., learning, fatigue, preceding responses; see Baayen et al., 2008) and hence allow us to assess possible practice effects (i.e., improvement in performance over the course of the experiment; Keuleers et al., 2012). Practice effects were tested by modeling trial order in two-way interactions with lexicality and with the psycholinguistic variable of interest (OLD20 or length according to the model). We note that the planned models on accuracy did not converge and are therefore not reported here. It is plausible that these models were too complex to be adequately supported by the collected data (Bates et al., 2015), given that our participants showed very high accuracy with little variance in their responses. Here we report data on log-transformed RTs.

The graphs show that the effects of OLD20, length, and trial order are modulated by lexicality (Fig. 11, panels a and b; Fig. 12). In line with the analyses on averaged data (see Fig. 10), non-lexical items with a denser orthographic neighborhood (i.e., those with lower OLD20) are harder to reject (e.g., Coltheart et al., 2001), whereas the opposite pattern is observed for lexical items (words with lower OLD20, i.e., a denser orthographic neighborhood, are easier to accept; Fig. 11, panel a). Item length also affects performance differently for lexical and non-lexical items, with a larger impact on the latter than the former (Fig. 12, panel a). Finally, practice effects are more evident for non-lexical stimuli than for lexical stimuli (panel b in Figs. 11 and 12). That is, over the course of the experiment, identifying non-lexical items becomes comparatively easier than identifying lexical items (Ferrand et al., 2018; Keuleers et al., 2010, 2012). The analysis also shows that participants become progressively less sensitive to OLD20 as the experiment proceeds (Fig. 11, panel c).

#### Effects of pseudoword type on pseudoword processing

**Fig. 13:**
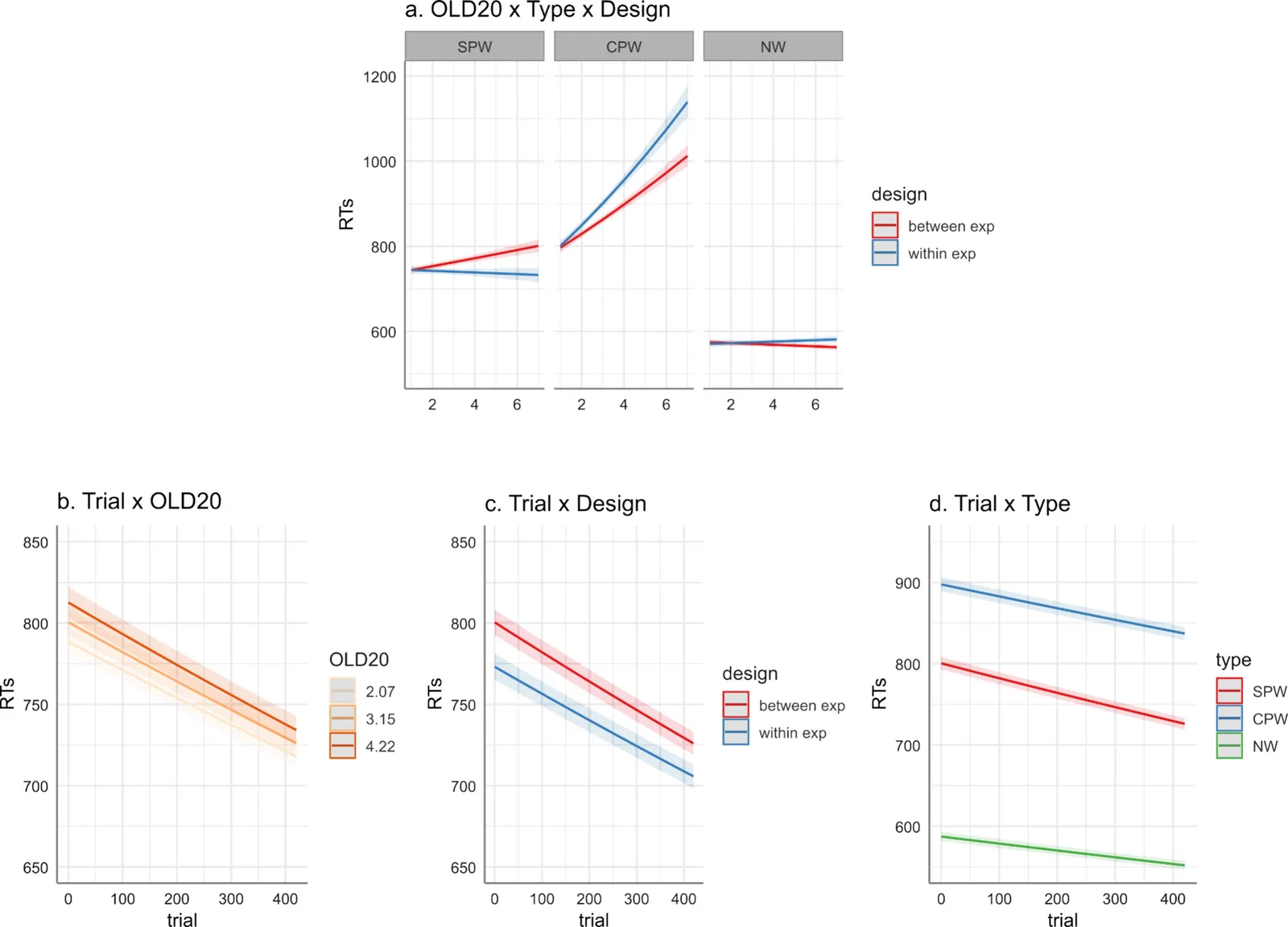
Results of the OLD20 model testing the effect of pseudoword type on pseudoword processing. Panel (**a**) shows the three-way interaction of OLD20 × type of pseudoword × design, where “between exp” represents RTs from Experiments 1, 2, and 3, while “within exp” represents RTs from Experiment 4. Other graphs represent the two-way interactions of Trial with OLD20 (**b**), design (**c**), and type (**d**). Abbreviations: SPW = simple pseudowords; CPW = complex pseudowords; NW = nonwords

**Fig. 14:**
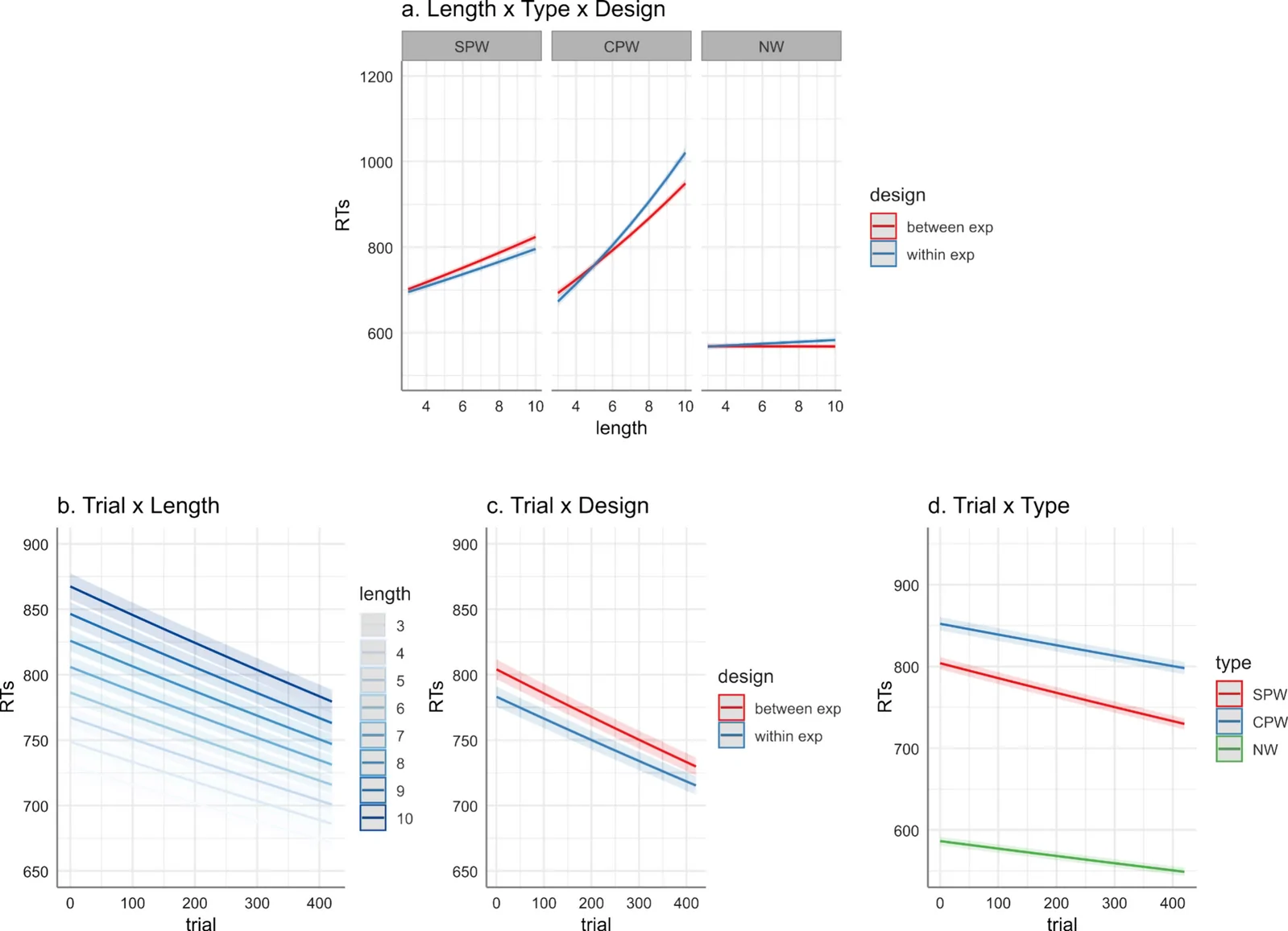
Results for the length model testing the effect of pseudoword type on pseudoword processing. Panel (**a**) shows the three-way interaction of length × type of pseudoword × design, where “between exp” represents RTs from Experiments 1–3, while “within exp” represents RTs from Experiment 4. Other graphs represent the two-way interactions of trial with length (**b**), design (**c**), and type (**d**). Abbreviations: SPW = simple pseudowords; CPW = complex pseudowords; NW = nonwords

To assess the effects of pseudoword type on pseudoword processing, we ran linear mixed-effects models for log-transformed RTs to non-lexical stimuli. The logistic models for accuracy failed to converge, so they are not reported here (see the Deviations section above). Each model in this section included one psycholinguistic variable of interest (either length or OLD20), item type (simple pseudowords vs. complex pseudowords vs. nonwords), design (within- vs. between-experiment), and trial order. We also tested all interactions among the psycholinguistic variable, item type and design, and allowed trial order to interact with each of these predictors. Random intercepts for participants and items were also included in all models. These models were supposed to disentangle how different non-lexical items and the context in which they were processed (i.e., without other types of non-lexical items in the between-experiment design condition [Experiments 1–3], or together with other types of non-lexical items in the within-experiment design condition [Experiment 4]), along with their psycholinguistic features (OLD20 or length; see Tables 10 and 11, respectively), shape correct pseudoword rejection, while also tracking practice effects across the experiment. The results are visualized in Figs. 13 and 14.

**Table 10:** Linear mixed-effects models with OLD20, type of non-lexical item, design, and trial order as predictors of time to rejection of pseudowords

	Sum Sq	Mean Sq	NumDF	DenDF	*F* value	Pr(>*F*)
OLD20_us	11.42	11.42	1	25783	198.83	<.001
Type	85.12	42.56	2	16856	740.88	<.001
Design	0.16	0.16	1	632297	2.85	.091
Trial	11.36	11.36	1	669371	197.68	<.001
OLD20_us × type	13.74	6.87	2	15837	119.57	<.001
OLD20_us × design	0.42	0.42	1	614167	7.34	.007
Type × design	1.01	0.51	2	615071	8.81	<.001
OLD20_us × trial	0.43	0.43	1	669447	7.45	.006
Type × trial	8.45	4.22	2	669566	73.53	<.001
Design × trial	0.39	0.39	1	668129	6.85	.009
OLD20_us × type × design	7.11	3.55	2	613532	61.88	<.001

**Table 11:** Linear mixed-effects models with length, type of non-lexical item, design, and trial order as predictors of rejection times to pseudowords

	Sum Sq	Mean Sq	NumDF	DenDF	*F* value	Pr(>*F*)
Length	90.96	90.96	1	32745	1583.44	<.001
Type	24.08	12.04	2	16691	209.57	<.001
Design	2.55	2.55	1	599960	44.38	<.001
Trial	5.00	5.00	1	670017	87.07	<.001
Length × type	71.80	35.90	2	16099	624.98	<.001
Length × design	3.76	3.76	1	580798	65.38	<.001
Type × design	2.28	1.14	2	582475	19.85	<.001
Length × trial	1.05	1.05	1	670121	18.30	<.001
Type × trial	10.17	5.09	2	670084	88.53	<.001
Design × trial	0.38	0.38	1	668555	6.59	.010
Length × type × design	7.39	3.70	2	580541	64.36	<.001

As expected, nonwords were the easiest to reject regardless of the context in which they were presented (e.g., as the only type of non-lexical items in Experiment 3 or intermixed with other types of pseudowords in Experiment 4), and their psycholinguistic features (OLD20 and length; see panel a in Figs. 13 and 14, respectively). Simple pseudowords were faster to reject than complex pseudowords (see the discussion on morpheme interference effect below; see also Crepaldi et al., 2010; Taft & Forster, 1975) but slower than nonwords. This aligns with the assumption of differential activation of the visual word system when stimuli contain familiar linguistic patterns, such as our simple pseudowords (e.g., Keuleers & Brysbaert, 2011; Ziegler et al., 1997), relative to stimuli that do not respect language graphotactics (i.e., nonwords). These results are also consistent with Barca and Pezzulo (2012), where mouse movements during classification of non-lexical items (similar to our simple pseudowords) were characterized by a biphasic process: mouse movements were initially attracted toward the “lexical” category, with a subsequent sharp deviation toward the “non-lexical category”; that is, participants initially committed to the lexical (i.e., incorrect) response and then subsequently switched their commitment to the (correct) non-lexical response. Conversely, classification of strings of letters similar to our nonwords was characterized by a straight trajectory toward the “non-lexical” category, with no initial bending toward the “lexical” category. In sum, our data align with literature suggesting that pseudowords undergo greater lexical interference effects than nonwords, resulting in slower reaction times for these categories of non-lexical items.

As for the effects of length and OLD20, these seem to differ between complex and simple pseudowords depending on the design factor. That is, when non-lexical types are intermixed (within-experiment condition, i.e., Experiment 4) the OLD20 interference effect is more evident for complex pseudowords, with less-word-like items being responded to more slowly. The effect seems to be reversed for simple pseudowords, with a facilitatory effect in the within-experiment condition (i.e., faster responding to less-word-like items) and interference effect (i.e., slower responding to less-word-like items) in the between-experiment condition (Fig. 13, panel a). The length effect shows a very similar pattern, with the only difference being that in simple pseudowords, we found an interference effect—that is, longer items were responded to more slowly in both types of design, albeit with a weaker effect in the within-experiment condition (Fig. 14, panel a). Arguably, for complex pseudowords, but not necessarily simple ones, classification requires participants to read the whole string to gather enough information to correctly categorize the item, thus resulting in longer reaction times.

**Fig. 15:**
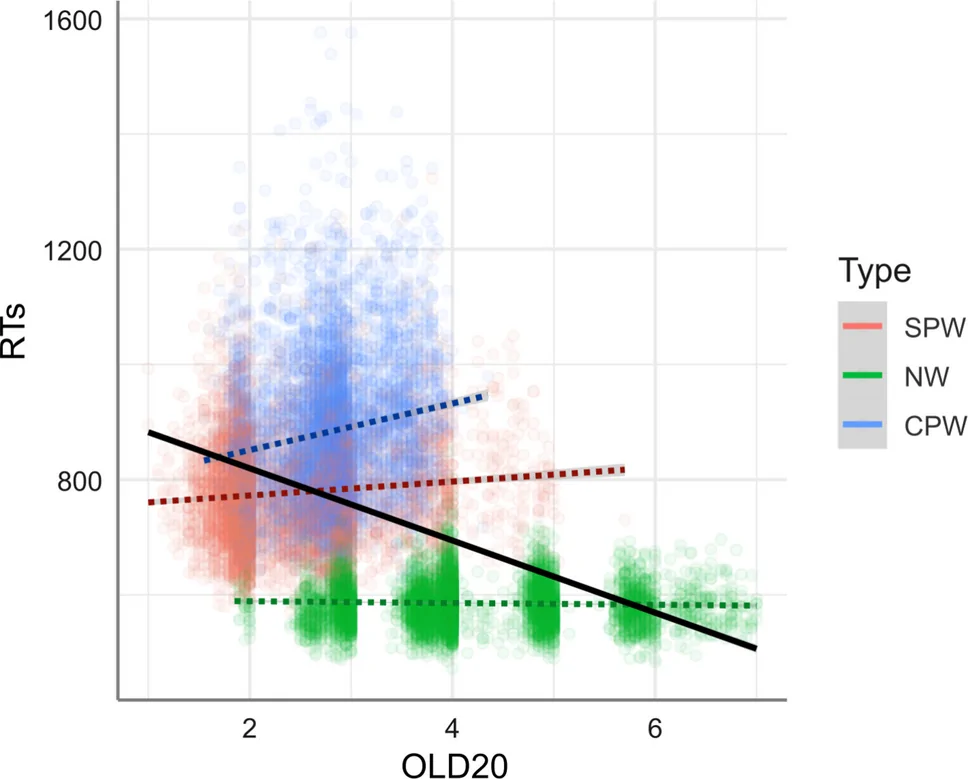
Reaction times for different types of non-lexical items as a function of OLD20. The lines represent separate linear regressions for each type of non-lexical item (dashed lines) or collapsing all types in a single non-lexical category (continuous line). Abbreviations: SPW = simple pseudowords; CPW = complex pseudowords; NW = nonwords

In the current analysis, OLD20 seems to have affected reaction times in a direction which is not compatible with what was observed in the previous lexicality analysis. In fact, when considering the three types of non-lexical items separately, OLD20 is associated with an interference effect (i.e., less-word-like stimuli being responded to more slowly), while when collapsing all of the non-lexical items in a single category OLD20 is associated with an overall facilitatory effect (i.e., faster responding to less-word-like stimuli). This apparent discrepancy can be explained as the result of nonwords having overall higher OLD20 and faster RTs than complex pseudowords, which conversely have lower OLD20 and slower RTs (Fig. 15). It is also worth noting that the discrepancy between the direction of the present OLD20 effect and the facilitatory effect reported by Hendrix and Sun (2021) might depend on the range of OLD20 values resulting from experimental control. The range of OLD20 for pseudowords in Hendrix and Sun (2021) spans from 1 to 6.42, which is close to what we have when we consider all non-lexical stimuli altogether. In fact, the range of OLD20 values for our non-lexical stimuli—for which we found a facilitatory effect as well—ranges from 1 to 7, with the maximum OLD20 value being different between types of items: 7 for nonwords, 5.7 for simple pseudowords, and 4.35 for complex pseudowords (Fig. 11, panel a). It is therefore possible that the direction of the effect of OLD20 on lexical decision times for non-lexical stimuli depends on the specific range of OLD20 that is taken into account.

Still, we highlight the striking similarities in our data between the effects of OLD20 and length (see also the *Effects of pseudoword type on word processing* section). This may indicate that, in the context of the present data, the OLD20 metric fails to gauge any variance other than that explained by length. In other words, OLD20 may in fact be a proxy measure of length. This is not entirely surprising, since collinearity between length and OLD20 was described as early as in the seminal work by Yarkoni et al. (2008). In our data, OLD20 and length show a high correlation (overall *r* =.65; lexical items *r* =.85; non-lexical items *r* =.60). Given the direction of the effects and the high correlations, we advise caution in interpreting the effects of these two measures as independent and suggest relying on length rather than OLD20 when using the current resource.

Finally, practice effects are found to modulate the impact of the other variables: over the course of the experiment, participants become less sensitive to OLD20 (Fig. 13, panel b) and item length (Fig. 14, panel b). The practice effect is more evident for pseudowords (i.e., simple and complex) than for nonwords (see panel d of Figs. 13 and 14), and in between-experiment design (i.e., when different types of non-lexical stimuli are not mixed with each other; see panel c of Figs. 13 and 14).

#### Effects of pseudoword type on word processing

To investigate how lexical decision performance for words is affected by different pseudoword types, we ran a series of linear mixed-effects models for log-transformed RTs to word stimuli. Logistic models for accuracy were omitted as they failed to converge. As predictors, in each model we considered a single psycholinguistic variable (OLD20, length, and zipf_us frequency; see Tables 12, 13, and 14, respectively), along with pseudoword type (representing the accompanying non-lexical stimuli, i.e., simple pseudowords versus complex pseudowords versus nonwords), trial order, and their two-way interactions. Random intercepts for participants and items were again included in all models.

**Table 12:** Linear mixed-effects model of response times for correct word identification with OLD20, pseudoword type, and trial order as predictors

	Sum Sq	Mean Sq	NumDF	DenDF	*F* value	Pr(>*F*)
OLD20_us	43.75	43.75	1	5499	738.55	<.001
PWtype	95.08	47.54	2	444481	802.53	<.001
Trial	8.96	8.96	1	443559	151.29	<.001
OLD20_us × PWtype	48.83	24.41	2	443705	412.13	<.001
OLD20_us × trial	0.25	0.25	1	443577	4.27	.039
PWtype × trial	12.18	6.09	2	443557	102.77	<.001

**Table 13:** Linear mixed-effects model of response times for correct word identification with length, pseudoword type, and trial order as predictors

	Sum Sq	Mean Sq	NumDF	DenDF	*F* value	Pr(>*F*)
Length	26.14	26.14	1	5330	442.05	<.001
PWtype	57.91	28.96	2	444235	489.64	<.001
Trial	5.95	5.95	1	443511	100.70	<.001
Length × PWtype	94.05	47.02	2	443559	795.16	<.001
Length × trial	0.21	0.21	1	443506	3.55	0.059
PWtype × trial	12.27	6.13	2	443519	103.70	<.001

**Table 14:** Linear mixed-effects model of response times for correct word identification with word frequency, pseudoword type, and trial order as predictors

	Sum Sq	Mean Sq	NumDF	DenDF	*F* value	Pr(>*F*)
zipf_us	249.71	249.71	1	6176	4244.76	<.001
PWtype	555.71	277.85	2	444737	4723.25	<.001
Trial	30.28	30.28	1	443801	514.65	<.001
zipf_us × PWtype	220.36	110.18	2	443843	1872.91	<.001
zipf_us × trial	6.97	6.97	1	443721	118.40	<.001
PWtype × trial	11.36	5.68	2	443733	96.59	<.001

**Fig. 16:**
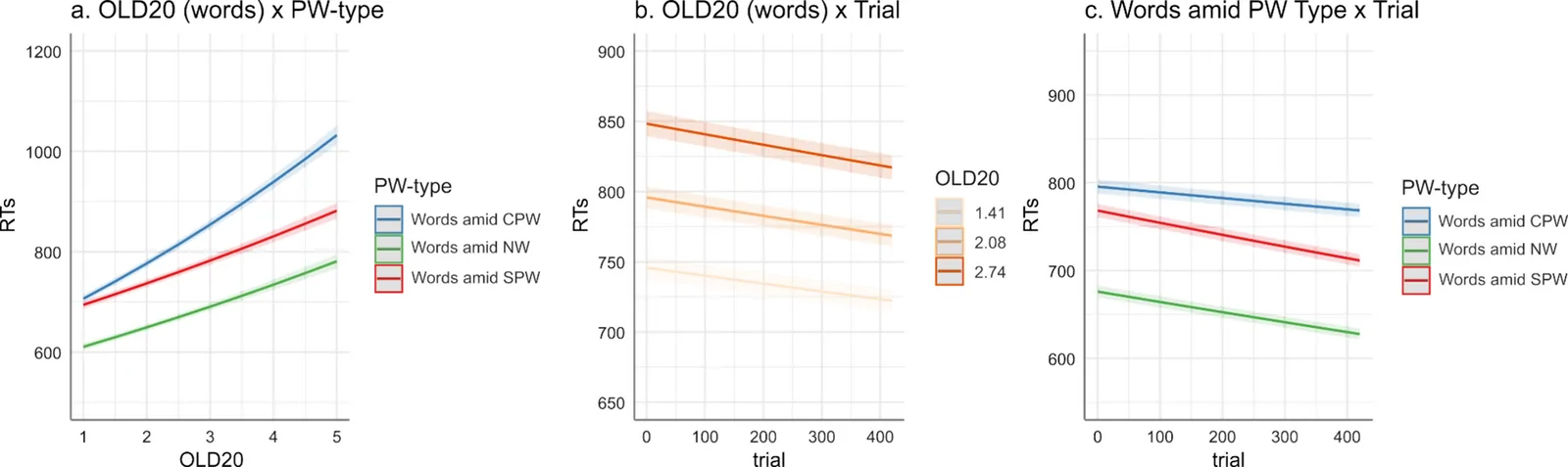
Results for the OLD20 model and significant interactions with pseudoword type and trial order on word processing

**Fig. 17:**
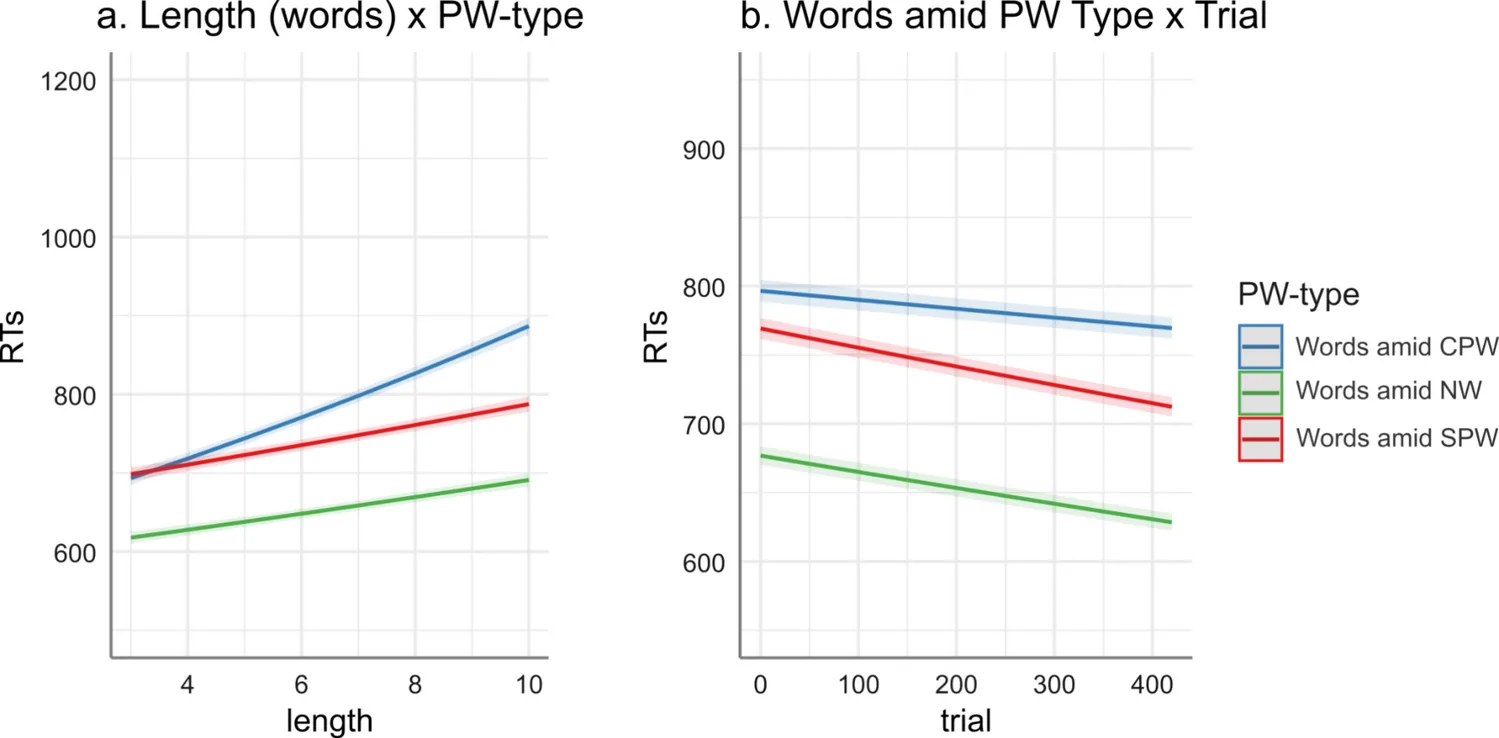
Results for the length model and significant interactions with pseudoword type and trial order on word processing

**Fig. 18:**
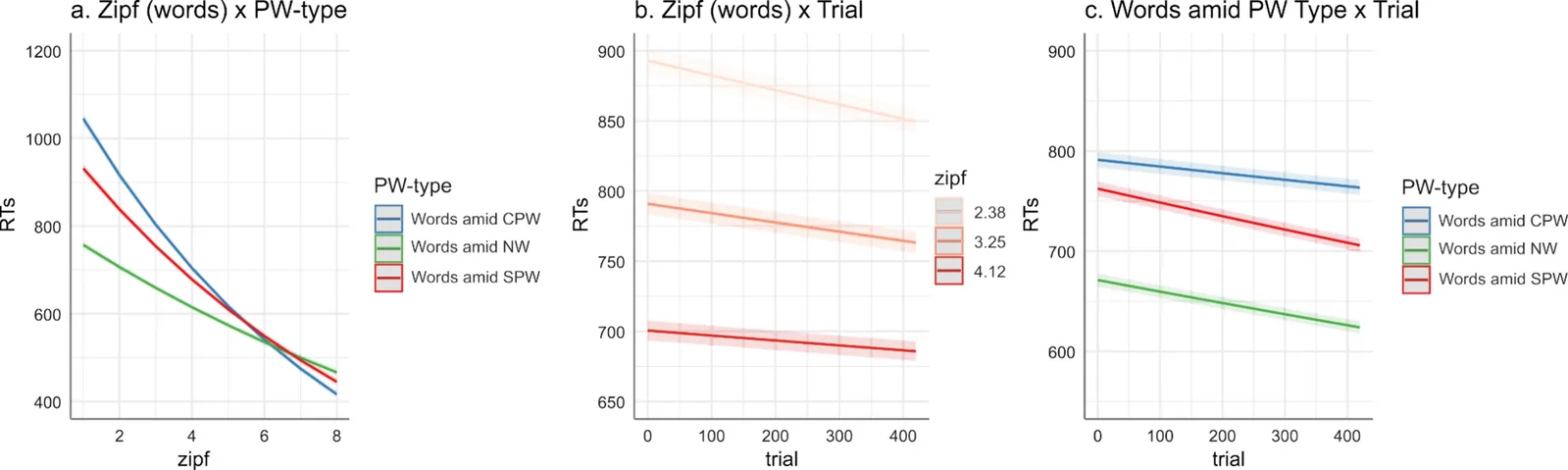
Results for the Zipf model and significant interactions with pseudoword type and trial order on word processing

Significant two-way interactions between the psycholinguistic variables of interest (OLD20, length, and frequency), pseudoword type, and trial order (i.e., practice effects) are visualized in Figs. 16, 17, and 18, respectively.

Our results confirmed the standard findings reported in the literature: participants responded faster to words with many orthographic neighbors (i.e., smaller OLD20; Andrews, 1992; Grainger et al., 1989), shorter words (Frederiksen & Kroll, 1976; Hudson & Bergman, 1985), and frequent words (Broadbent, 1967; Brysbaert et al., 2018). However, these effects on words were modulated by the presence of different types of pseudowords, with the most evident effect on words presented amid complex pseudowords (see panel a of Figs. 16, 17, and 18). That is, when words were embedded in lists containing complex pseudowords (i.e., in Experiment 2), the effects of OLD20, length, and frequency were more pronounced. Such a pattern is inconsistent with our predictions and might result from the increased lexical competition occurring in this condition. In fact, it is plausible that morphologically complex pseudowords are decomposed into morphemic constituents (stem and affix), making them directly available for processing (Longtin & Meunier, 2005; Rastle & Davis, 2008). For example, the pseudoword “quickify” may activate the representations of “quick” and “-ify” and in turn evoke a plausible semantic interpretation of the whole word (e.g., to make something quicker; see Feldman et al., 2009). This decomposition process may increase the overall lexical and semantic activation within the system, as even non-lexical items become interpretable, and thus similar to existing words. As a consequence, words that are a priori more demanding to process (i.e., longer words, less frequent words, and those with fewer orthographic neighbors) may become even more so: the morphological decomposition of complex pseudowords and the task requirement for rejecting them amplifies lexical competition, resulting in stronger effects of lexical variables on word recognition.

Such dynamics within the linguistic system would impact the participants’ decision processes, with morphologically complex pseudowords, containing meaning-bearing units that produce greater overall activation of the lexicon (see for instance Grainger & Jacobs, 1996), essentially increasing uncertainty in lexical decisions. This may result in participants setting decision thresholds (Perea et al., 2004) so as to maximize their sensitivity to lexical properties of word stimuli to provide their response. That is, in dealing with lists composed of words and morphologically complex pseudowords, participants may adjust their strategy so as to anchor “word” responses to short, familiar stimuli.

On the other hand, the greater length effect for words included in lists containing morphologically complex pseudowords may be because this latter class of non-lexical stimuli forces participants to serially process each letter string in the list. Indeed, since a stimulus like “healthize” (health+ize) is non-lexical and “healthy” (health+y) is, in fact, a word, fast parallel recognition of lexical elements (Ellis, 2004; Ellis et al., 1988) is not sufficient to tease apart words from pseudowords, and hence it is not a viable strategy for solving the task. The need for participants to resort to serial reading of incoming stimuli to perform lexical decisions would therefore make length effects comparatively more evident in lists containing complex pseudowords than in lists including morphologically simple pseudowords or nonwords.

Regarding practice effects, we observed significant interactions between trial order and two psycholinguistic variables of interest (OLD20 and frequency; see panel b of Figs. 16 and 18). Through visual inspection of these interactions, it is observed that the effect of practice is the strongest for words with fewer orthographic neighbors (larger OLD20; Fig. 16, panel b), low-frequency words (lower Zipf; Fig. 18, panel b), and words accompanied by simple pseudowords (panel c of Figs. 16 and 18, panel b of Fig. 17).

#### Ancillary analysis: Effect of pseudoword type on morphological effect in words

To investigate how the different pseudoword types used across the four experiments affect lexical decision performance differently for simple vis-à-vis complex words, we ran a linear mixed-effects model for log-transformed RTs using word morphology and experiment as predictors, together with their interaction (Table 15).

**Table 15:** Linear mixed-effects models with word morphology and experiment as predictors for response times in correct word identification

	Sum Sq	Mean Sq	NumDF	DenDF	*F* value	Pr(>*F*)
Morphology	0.81	0.81	1	4262	13.37	<.001
Exp	1,607.00	535.67	3	585260	8792.53	<.001
Morphology × Exp	90.66	30.22	3	592409	496.06	<.001

**Fig. 19:**
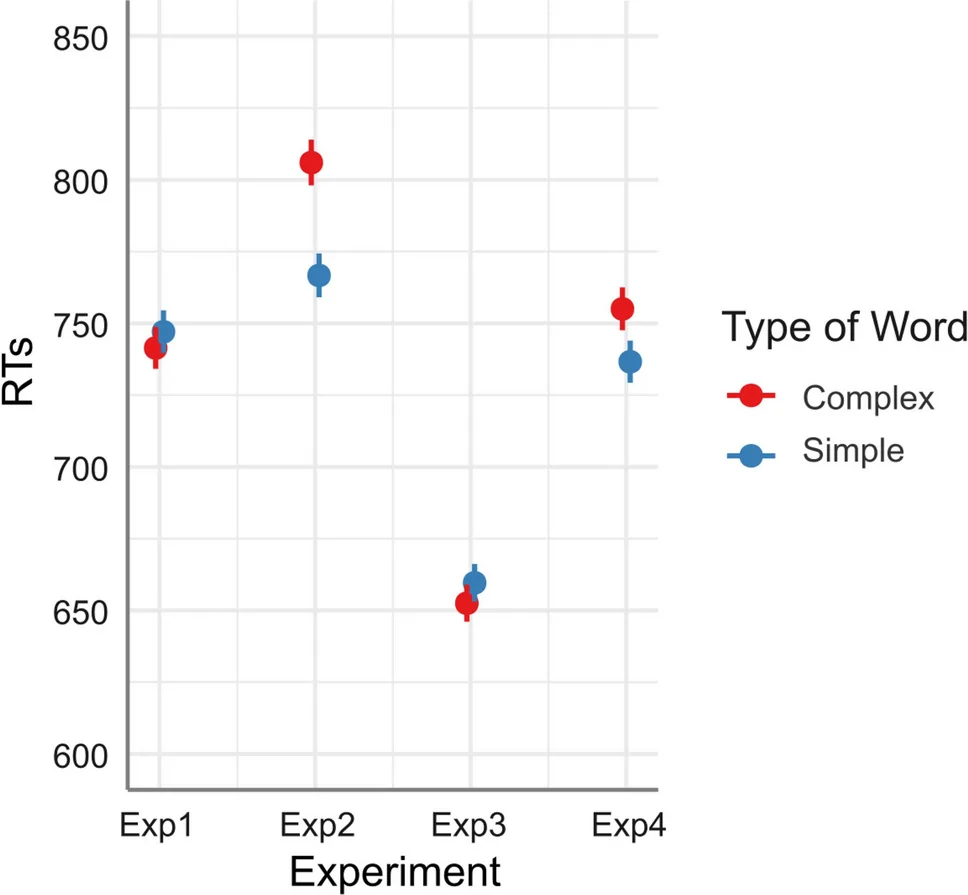
Results of the ancillary analysis on response times for simple vis-à-vis complex words across the four experiments. Non-lexical stimuli presented along with words were simple pseudowords in Experiment 1, complex pseudowords in Experiment 2, and nonwords in Experiment 3, while in Experiment 4 they were a balanced combination of all three types of non-lexical stimuli (i.e., one third simple pseudowords, one third complex pseudowords, one third nonwords)

We observe that the pseudoword type included in each experiment affects the impact of words’ morphological complexity (Fig. 19). Morphologically simple words elicited faster responses than complex words when presented in a list including complex pseudowords (Experiments 2 and 4; both *p* <.001). Interestingly, this effect is reversed (although the overall magnitude of this reversal is marginal) when words are presented along with nonwords (Experiment 3; *p* <.05, BF_10_ = 0.086^8^), and nonsignificant when words are presented with simple pseudowords (Experiment 1; *p* =.23).

Taken together, these results point to a list effect associated with the degree of structure (i.e., presence of evident sublexical patterns that are typical of English) of the included pseudowords. The more structured the pseudowords are in terms of graphotactic properties—ranging from nonwords (least structured), to simple pseudowords, and finally to complex pseudowords (most structured)—the more participants appear to rely on sublexical (i.e., not tapping into lexical processing) elements when processing lexical stimuli. This results in longer processing times for complex words, which notably embed morphemic units. In Experiment 2 (where complex pseudowords were the only type of non-lexical items) and, to a lesser extent, in Experiment 4 (where complex pseudowords were presented along with other types on non-lexical item), word identification was made more difficult by the presence of morphemic units, as decomposition would be encouraged by the prevalence of morphological information across different types of stimuli (Amenta et al., 2025; Longtin & Meunier, 2005; Marslen-Wilson et al., 2008). In contrast, in the absence of complex pseudowords (i.e., Experiments 1 and 3), the lexicality judgement appears to depend less and less on sublexical cues, thus attenuating or even reversing the morphology effect: indeed, in Experiment 3 the presence of nonwords, i.e., the least word-like stimuli, may by contrast facilitate the “word” decision for morphologically complex items, i.e., the most “lexically rich” stimuli.

## General discussion

We have conducted a lexical decision megastudy, which we named the English Pseudoword Lexicon Project (EPLeP), to produce and validate a comprehensive behavioral resource designed for psycholinguistic research that specifically (although not exclusively) focuses on non-lexical stimuli, namely pseudowords and nonwords. This resource addresses a critical gap in the literature, as previous megastudies conducted in different languages (see Table 1) focused almost exclusively on words. Here we brought pseudowords into the spotlight, by manipulating the orthographic and morphological features of non-lexical stimuli and by relating such features to behavioral performance. We created a set of 4,523 morphologically simple (yet graphotactically legal) pseudowords, 4,498 morphologically complex (legal) pseudowords and 4,475 nonwords (i.e., letter strings that violate English graphotactic rules). These stimuli were included in a lexical decision task together with morphologically simple and complex words. We tested lexical decision times for these three types of non-lexical stimuli while keeping them separate (Experiments 1–3) and intermixed (Experiment 4), using the same set of words.

First of all, across all experiments, split-half reliability for morphologically simple and morphologically complex pseudowords (fluctuating around .60 and .70 for raw correlations and around .80 for correlations with Spearman–Brown correction) turned out to be comparable to that of the data reported by Ferrand et al. (2010, 2018). Contrary to our expectations, reliability turned out to be the lowest for nonwords (estimated at .17 for raw correlations and .29 following Spearman–Brown correction). Although the lack of previous data in the literature limits any conclusive claim in this regard, we hypothesize that this effect might be due to nonwords being associated with the highest performance: the few observed errors and variations in responses may be due to idiosyncratic factors (e.g., occasional lapses of attention) that are not related to the systematic properties of stimuli and therefore hardly generalizable across participants. On the other hand, validity analyses reported significant positive correlations between the performance in our study and data from the British Lexicon Project (Keuleers et al., 2012) for shared items across the two megastudies. Taken together, reliability and validity results indicate that the EPLeP data are useful and aligned with previous literature (Ferrand et al., 2010; Keuleers et al., 2012; Schröter & Schroeder, 2017), showing them to be in line with the literature standards for lexical decision megastudies.

Since one of the main aims of the present megastudy is reassessing the role of non-lexical stimuli as tools for psycholinguistic enquiry (see also Yap et al., 2015), we conducted a series of analyses (*lexicality analyses*), in which we compared performance during processing of lexical versus non-lexical stimuli. Contrary to our expectations, we observed a reversed lexicality effect on RTs, with non-lexical items being responded to faster than lexical items. According to Coltheart et al. (1977), participants make “no” responses via extra-stimulus information, using a flexible deadline criterion. If lexical information (in terms of the sum of activation in the orthographic lexicon) is high early in processing, the deadline can be increased. If there is very little activation in the orthographic lexicon, the deadline can be shortened (Coltheart et al., 1977, 2001; Grainger & Jacobs, 1996; Perea et al., 2005). According to this deadline account, non-lexical items (e.g., in Experiment 3) that are very nonword-like, composed of random strings of letters, result in very little activation in the orthographic lexicon and are easy to reject. A similar explanation may apply to Experiment 1, in which the simple pseudowords may have also been relatively easily identifiable, although the lexicality effect was not significant (*F*(1, 8,194.4) = 0.184, *p* = 0.668). It is plausible that such reversed effect occurs only in specific experimental contexts and task demands (e.g., such as speed–accuracy trade-off or proportion of lexical vs. non-lexical items; see Scaltritti et al., 2025; Wagenmakers et al., 2004; Zevin & Balota, 2000) which cause participants to make qualitative changes in their lexical decision-making process, that best suit the demands of the situation (see also Borowsky & Besner, 2006; Plaut & Booth, 2000). We therefore speculate that when nonwords are highly implausible or perceptually distinctive (e.g., “reknrhhh” in Experiment 3), participants may rely more heavily on rapid rejection strategies, resulting in attenuated (or even reversed) lexicality effects. In addition, we highlight that in all experiments, half of the word stimuli were morphologically complex. This may have resulted in pseudowords being particularly easy to distinguish from lexical items overall (as the global accuracy levels testify), especially when unattested stimuli were morphologically simple (i.e., Experiment 1) or graphotactically implausible (i.e., Experiment 2). Further evidence supporting this hypothesis comes from Experiment 2, in which morphological cues were available in both words and pseudowords, and in which the lexicality effect in the usual direction emerged.

In addition, within *lexicality analyses*, we quantified lexicality modulation of the effect of orthographic variables (i.e., stimuli length and orthographic neighborhood, operationalized with OLD20), as well as in practice-related phenomena. In these analyses, we compared words to non-lexical stimuli without taking into account morphological complexity or compliance with orthotactic rules (i.e., morphologically simple pseudowords, morphologically complex pseudowords, and nonwords were all labeled as “non-lexical” in the context of these analyses). As expected, we observed a specular effect of OLD20 between lexical and non-lexical items: a high number of orthographic neighbors facilitates “word” responses and inhibits “nonword” responses. Conversely, contrary to our expectations, the effect of length was in the same direction for words and pseudowords (i.e., longer reaction times for longer stimuli), even though it was more pronounced for pseudowords than for words. Although this pattern of data is in line with previous literature (Balota et al., 2004; Hudson & Bergman, 1985; O’Regan & Jacobs, 1992; Whaley, 1978; Yap et al., 2015), it is worth mentioning that there is ample debate in the literature on length effects in orthographic processing (Baayen, 2005; Frederiksen & Kroll, 1976; Hudson & Bergman, 1985; New et al., 2006; O’Regan & Jacobs, 1992; Richardson, 1976), especially in relation to lexicality (Hendrix & Sun, 2021). As far as the present data are concerned, we suggest that the overall linear effects of length reflect early mechanisms of visual uptake that are common to all incoming orthographic stimuli regardless of their lexicality. Still, this does not in principle exclude the possibility that the effect of stimulus length on performance might be fruitfully captured by nonlinear associations (which remain beyond the scope of the present work). As for the effect of practice, we report a more prominent increase in performance for pseudowords than words and (across lexicality) a steeper reduction in RTs for more word-like stimuli. Taken as a whole, these results provide a broad overview of lexicality effects in orthographic processing: a specular effect of OLD20, a comparable (although stronger for pseudowords) effect of word length, and a comparable (although—again—stronger for pseudowords) effect of practice on performance overall.

Yet, critically, pseudowords are not all alike. In the *effect of pseudoword type on pseudoword processing* analyses, we manipulated morphological complexity or compliance with orthotactic rules by comparing lexical decision performance for morphologically simple pseudowords, morphologically complex pseudowords, and nonwords. We observed the effects of OLD20 and length to be almost null in nonwords, while being more pronounced for complex pseudowords than simple ones. Overall, nonwords were associated with faster rejection as compared with simple and complex pseudowords. This is in line with the Balota and Chumbley (1984) model (see also Balota & Spieler, 1999), which suggests that lexical decision performance often involves two steps: a “familiarity evaluation” (see Ablinger & Domahs, 2009; Cohen & Dehaene, 2000, for neuropsychological evidence in the same direction) that can return either a high score (i.e., for words, thus “word” response can be made) or a low score (i.e., for pseudowords, as they are not attested in the lexicon, preceding the “nonword” response). This intermediate score initiates (if necessary) a second process which is essentially a spell-checking process. Since nonwords used in Experiment 3 are particularly nonword-like, the second process is omitted and thus the entire lexical decision-making takes shorter time (as seen from the fast response times for nonwords in Experiment 3). The second process appears to be more relevant when simple and complex pseudowords are encountered, as their word-likeness slows down item rejection. It is also worth noting that the OLD20 pattern for morphologically simple and complex pseudowords suggests a rather counterintuitive facilitatory effect of orthographic neighborhood on rejection times. Although this might be due to our focus on clusters of OLD20 that have a narrower range than previously explored (Hendrix & Sun, 2021), we cannot formally exclude the possibility that our results on OLD20 simply represent a reverberation of length effects, due to the high correlation between these two variables. As outlined in the previous sections, we invite future users of this resource to consider the effect of the OLD20 metric with caution.

Apart from the effects on graphotactically implausible items, we also replicated a standard finding of non-lexical items undergoing morphological decomposition (Perea et al., 2005; Whaley, 1978; Yap et al., 2015). That is, rejection of pseudowords with a morphological structure (i.e., embedding a stem and an affix) requires longer reaction time than pseudowords without a morphological structure (Amenta et al., 2025; Beyersmann et al., 2015, 2020; Crepaldi et al., 2010; Hasenäcker et al., 2016; Taft & Forster, 1975). This is particularly evident in Experiment 4, in which complex pseudowords are responded to more slowly than simple pseudowords and nonwords. This effect could be explained in terms of the ability of morphological units to activate lexical representation. That is, the mere presence of a possible stem and a possible affix (i.e., a morphological structure) is sufficient to hinder the recognition process, inducing “yes” responses even for unattested strings, resulting in longer response times and more false positives. Moreover, a legal combination of stem and affixes merged into a novel word makes readers attempt meaning induction via a combinatorial process (Amenta et al., 2025; Marelli & Baroni, 2015), hinting at a productive use of morphological information. As a consequence, the rejection of pseudowords containing morphemes is more cognitively demanding than that of simple pseudowords, a finding also confirmed in eye-tracking studies (Beyersmann et al., 2020; Lázaro et al., 2023). This is consistent with findings by Muncer et al. (2014) as well, and supports the view that morphologically complex stimuli are decomposed at an early, relatively automatic stage of visual word recognition (Rastle & Davis, 2008; Rueckl & Aicher, 2008). Broadly speaking, these results suggest that pseudoword processing is not qualified merely by early orthographic (and sublexical, see Coltheart et al., 2001) mechanisms: if this was the case, we would have found no differences across the different stimulus types. Rather, our data clearly indicate that pseudoword processing taps into morphological—and arguably lexical-semantic—mechanisms (Bonandrini et al., 2023), even though for morphologically complex stimuli the relevance of orthographic versus semantic mechanisms and how they unfold in the temporal domain is still a matter of debate (Amenta et al., 2015, 2020; Amenta & Crepaldi, 2012; Beyersmann et al., 2020; Feldman et al., 2009; Rastle et al., 2004).

In line with these conclusions, in the *effect of pseudoword type on word processing* analyses, pseudowords were shown to affect word processing by virtue of the extent to which they engage lexical-semantic processing. This is consistent with previous data suggesting that performance in a lexical decision task depends on how difficult it is to tease apart lexical stimuli from non-lexical ones (Lupker & Pexman, 2010). In the context of our megastudy, more structured non-lexical stimuli (i.e., complex pseudowords > simple pseudowords > nonwords) yielded slower responses for words. Moreover, relative to the other pseudoword types, morphologically complex pseudowords strengthened the effects of OLD20, length, and frequency in word processing; conversely, nonwords reduced the magnitude of the effects of length and frequency. We interpret these results as due to the fact that, given their parsability in existing stems and suffixes, morphologically complex pseudowords are particularly structured in terms of morphosemantic information. Thus, in a list containing this type of pseudowords, psycholinguistic properties become very salient during the process of identification of lexical stimuli as such. Conversely, since nonwords hardly contain structured elements at all, lexical decisions for words in a list containing these types of pseudowords requires lower reliance on deep lexical-semantic psycholinguistic features. Furthermore, as our study controlled for morphological complexity in words, we could qualify performance for simple and complex words, showing that the type of accompanied pseudowords affected the relative processing of these two word types (see *Ancillary analysis*: *Effect of pseudoword type on morphological effects in words*). Simple words are generally processed faster than complex ones. However, this difference disappeared when words were presented along with simple pseudowords (i.e., Experiment 1), was reversed (even though the effect turned out to be marginal) when they were presented with nonwords (i.e., Experiment 3), and was most pronounced when the non-lexical items included a morphological structure (i.e., Experiment 2 and, to a lesser extent, Experiment 4). This pattern indicates that pseudowords can affect the degree of sensitivity to morphological cues during word processing in a lexical decision task. In particular, a list containing morphologically complex pseudowords as non-lexical stimuli triggers an in-depth analysis of word stimuli which entails appreciation of morphological cues; conversely, lists containing exclusively simple pseudowords or nonwords foster a shallower analysis of lexical stimuli, for which morphological decomposition is simply not necessary.

Taken as a whole, the results on the effect of pseudoword type on words adds to the literature on the long-standing issue of linguistic context and list effects (e.g., Danelli et al., 2015; Dorfman & Glanzer, 1988; Ferrand & Grainger, 1996) in language tasks, by emphasizing the fact that pseudowords dramatically affect how lexical decision is carried out. It is indeed evident that the nature of non-lexical items drives qualitative changes in participants’ behavior to best suit the demands of the task (Borowsky & Besner, 2006; Lupker & Pexman, 2010; Plaut & Booth, 2000; Yap et al., 2010), which may be associated with deeper (morphologically complex pseudowords) or shallower (nonwords) analysis of the incoming stimuli. If, on the one hand, this emphasizes the—at this point almost trivial—importance of controlling for stimuli list in a lexical decision task, on the other hand it highlights the not-so-trivial fact that one inescapable component of lexical decision (i.e., pseudowords), which has typically been considered to be of no interest, dramatically affects processing of the type of stimulus which has typically been considered “of interest” (i.e., words). This signals the need for a “contextual reframing” of our knowledge of the cognitive underpinnings of word processing: everything we know about words that comes from the lexical decision literature is inherently a function of the features of the pseudowords that have been used in each given study (see also Martínez-Tomás et al., 2025).

Overall, our results align with predictions of the parallel distributed processing (PDP) model proposed by Plaut and Booth (2000), which assumes that lexical decision performance is driven by semantic rather than purely lexical processing. According to this model, word–nonword decisions are based on the calculation of a unidimensional measure known as “semantic stress.” Words generate higher levels of stress because their orthographic structures activate semantic units more than nonwords do. Therefore, a criterion on the semantic stress dimension is assumed to allow words to be successfully discriminated from nonwords. The model also assumes that pseudowords that contain more structured elements would generate higher levels of semantic stress, causing the criterion to become more conservative, thus prolonging both word and pseudoword processing (i.e., latencies). In our case, higher levels of semantic stress produced by more structured pseudowords (e.g., complex pseudowords) also resulted in more pronounced differences in response speed between simple and complex pseudowords. On the other hand, nonwords (i.e., Experiment 3) do not contain any structured elements that resemble real English words, thus producing no semantic stress. The response criterion for yes/no decisions is liberal, resulting in overall faster response times and no difference in responding to simple and complex words. It is thus possible that gradation of semantic stress produced by pseudowords, from lower to greater, results in a difference in processing between simple and complex words from lower to higher, respectively (i.e., Experiment 1–3).

In an even broader perspective, our data show that pseudoword/nonword response data help provide meaningful and complementary insight into the lexical processing architecture (Yap et al., 2015). In this regard, the EPLeP adds to a growing body of literature advocating a more central role for pseudowords in psycholinguistics. Indeed, recent literature has highlighted the fact that pseudowords may trigger lexical-semantic processing more than previously anticipated (Bonandrini et al., 2023; Gatti et al., 2023; Günther et al., 2026; Hendrix & Sun, 2021; Hsieh et al., 2024), which opens up the possibility of adopting pseudowords as a tool to study the effect of lexical-semantic processing (which has typically been a prerogative of words), even in the absence of visual familiarity with the stimuli. In this context, the introduction of a large data resource providing a window into pseudowords appears timely and much needed. Here, with EPLeP, we present a broad corpus of data and tentative directions for interpretation which we hope will inform future psycholinguistic research.

As a final remark, we emphasize that, even though the present study explicitly focuses on English as a linguistic case study, the cognitive implications of the reported interaction between pseudoword reading and broader lexical-semantic processing phenomena are hardly limited to English-speaking populations. Indeed—we believe—our results ultimately tap into the broad issue of the architecture of the lexical-semantic system in a language-independent fashion. Nevertheless, the peculiarities of the English language, together with growing awareness of the pitfalls of anglocentrism in psycholinguistic research (e.g., Brysbaert, 2026; Share, 2008), encourage the exercise of caution in drawing conclusive claims. Undoubtedly, cross-linguistic evidence will be required to ascertain the relative relevance of structural orthographic and morphological components of the reference language in giving rise to the phenomena we highlighted here. In particular, it would be interesting to explore the extent to which the present results can be replicated in languages characterized by greater orthographic transparency (e.g., Italian, Spanish), in languages characterized by agglutinative morphology (e.g., Turkish, Japanese, Korean), and in languages in which word boundaries are more difficult to define (i.e., Mandarin).

## Conclusions

The English Pseudoword Lexicon Project (EPLeP) contains 13,496 pseudowords distributed across three categories (simple pseudowords, complex pseudowords, nonwords). Results indicate that such pseudoword types differ in the extent to which they engage lexical orthographic and morphological mechanisms: morphologically complex pseudowords trigger the deepest level of analysis, and nonwords the shallowest. Crucially, EPLeP data highlight that the effect of pseudoword types reverberates onto how words are processed as well. Researchers will be able to use EPLeP to answer theoretical questions, test model predictions, validate automated systems, select experimental or control stimuli, and support their research projects. One particularly useful feature of the EPLeP is the inclusion and systematic control of the presentation of the three most commonly used types of pseudowords (in separate experiments), which, together with a large set of items, represents comprehensive coverage of potential variables of interest. In addition to psycholinguistic research, EPLeP can be applied to educational and clinical fields/research as either a resource for materials or as a means for investigating the effects of psycholinguistic variables on language learners and clinical populations.

### Output data structure

The data for the words and the pseudowords are available online (https://osf.io/cz9rh/) as separate spreadsheets (in .csv format) named *Raw data* (one for each experiment), *Mean data per stimulus*, and *Participants*. These files will include all raw data per trial across four experiments and enable everyone to run analyses as if they had collected the data themselves. Words in brackets represent corresponding variable names in the released data files.

Each *Raw data* file contains the following information:

**Participant (id)**: identification number of the participant
**Block (block)**: the number of the block in which the item was presented for each participant, in range 1–4
**Trial number (trial):** number representing the order in which trials have been presented
4) **List number (list)**: number of the list of stimuli participant has been exposed to, in range 1–22
**Stimulus (stimulus)**: the stimulus (trial) presented on the screen
**Lexicality (lexicality)**: whether the stimulus is a simple word (SW), a complex word (CW), a simple pseudoword (SPW), a complex pseudoword (CPW), or a nonword (NW)
**Catch (catch)**: it signals whether that the stimulus is used as a catch trial for performance evaluation (“catch”)
**Response provided (response)**: the participant’s response to the stimulus: word (W), pseudoword (PW), or time-out (T)
**Accuracy (accuracy)**: the accuracy of the participant’s response: correct (1), incorrect (2), time-out (3)
**RT raw (rt)**: RT on the trial without cleaning/trimming

The second file, titled *Mean data per stimulus*, contains the complete lists of stimuli and their related information in aggregated form. Note that some of them will have a higher number of observations because they will be used in multiple experiments. For instance, certain simple pseudowords used in Experiment 1 will be reused in Experiment 4. This spreadsheet includes the following columns:

1. **Study (exp)**: code of the study in which the data are collected
2. **Stimulus (stimulus)**: the stimulus for which the data have been collected
3. **Lexicality (lexicality)**: whether the stimulus is a simple word (SW), a complex word (CW), a simple pseudoword (SPW), a complex pseudoword (CPW), or a nonword (NW)
4. **Length (length)**: length of the item in characters
5. **Syllables (syllables)**: number of syllables in the item (computed using the *nsyllable* package for R following English dictionary)
6. **Stem (stem)**: stem of the stimulus (applies to complex stimuli only)
7. **Suffix (suffix)**: suffix of the stimulus (applies to complex stimuli only)
8. **Frequency US (zipf_us)**: Zipf frequency of word from SUBTLEX-US
9. **Frequency UK (zipf_uk)**: Zipf frequency of word from SUBTLEX-UK
10. ** Stem frequency US (stem_zipf_us)**: Zipf frequency of stem from SUBTLEX-US (applies to complex pseudowords only)
11. ** Stem frequency UK (stem_zipf_uk)**: Zipf frequency of stem from SUBTLEX-UK (applies to complex pseudowords only)
12. ** OLD20 US (OLD20_us)**: the average orthographic Levenshtein distance of the 20 most similar words in the US corpus
13. ** OLD20 UK (OLD20_uk)**: the average orthographic Levenshtein distance of the 20 most similar words in the UK corpus
14. ** Prevalence (prevalence)**: word prevalence from Brysbaert et al., 2019
15. ** Pseudo-prevalence (pseudo_prevalence)**: number of false alarms for each non-lexical item
16. ** Ntrials (ntrials)**: total number of observations for the item
17. ** Accuracy (accuracy)**: mean accuracy for the item (based on all collected responses across participants), after exclusion of the invalid data points
18. ** Response time (rt)**: mean RT of the correct trials for the item (without invalid data points)
19. ** RT SD (rt_sd)**: standard deviation of the RTs for the item (without invalid data points)

The third file, titled *Participants*, contains demographic information of the participants. We note that no further analyses based on participants’ characteristics are provided. However, we encourage other researchers to merge this information with raw data using the participant’s unique identification number. This file will include the following information:

1. **Participant (id)**: identification number of the participant
2. **Age (age)**: participant’s age
3. **Sex (sex)**: participant’s sex (from Prolific’s demographic data)
4. **Ethnicity (ethnicity)**: participant’s ethnicity (from Prolific’s demographic data)
5. **Nationality (nationality)**: participant’s nationality (from Prolific’s demographic data)
6. **Country of birth (country_birth)**: participant’s country of birth (from Prolific’s demographic data)
7. **Country of residence (country_residence)**: participant’s country of residence (from Prolific’s demographic data)
8. **First language (first_language)**: participant’s first language (from Prolific’s demographic data)
9. **Student/employment status (work_status)**: participant’s student/employment status (from Prolific’s demographic data)
10. ** Highest education level (education)**: highest education level achieved by the participant

## Supplementary Information

Below is the link to the electronic supplementary material.

Supplementary file1 (DOCX 2359 KB)

## Data Availability

The stimuli for all experiments and collected data reported in this manuscript are available on the OSF page of the project (https://osf.io/cz9rh/).

## Footnotes

1 In this paper, we define “nonword” as a sequence of letters that does not form a valid word in a given language, given its graphotactics (i.e., English in this study; such as “btnick”; Schuster & Lahiri, 2019), and “pseudoword” as a sequence of letters that could validly form a word in that language but is not reliably attested in current usage (such as “blick” or “blickful”).

2 A megastudy approach has been adopted in crowdsourcing projects assessing participants’ word knowledge in English (Mandera et al., 2020), Spanish (Aguasvivas et al., 2018), Catalan (Guasch et al., 2022), Dutch (Brysbaert et al., 2019), and Italian (Amenta et al., 2025).

3 We opted for suffixed complex words rather than prefixed ones, as research on morphological decomposition has focused primarily on suffixed words (e.g., Marslen-Wilson et al., 2008; Rastle et al., 2004; Rastle & Davis, 2008).

4 This step was carried out in the Python version of Wuggy, with the “orthographic english” reference set and the “generate_classic” function with 20 items as output.

5 We note that some simple pseudowords have onsets or endings resembling existing affixes (e.g., “prerool”), but these stimuli are not morphologically parsable and cannot be segmented into known morphemes, which aligns with our working definition. Therefore, we have opted to keep them in the set, as they contribute to a varied and rich resource, and could be useful for future applications and reanalyses.

6 Since no standard deviation was obtained for three-letter stimuli, we imputed it by leveraging the linear relation (*t* = 6.860, *p* = 0.001) between stimuli length and standard deviation of the OLD20 in stimuli of 4–10 letters.

7 All experimental lists can have a variability of ±1 stimulus.

8 Upon suggestion from one of the reviewers, we conducted additional Bayesian exploration of this result to further validate its statistical meaningfulness (i.e., especially in relation to the relatively large sample size we adopted).

## Code availability

The code used to generate stimuli and to administer the experiments is available on the OSF page of the project (https://osf.io/cz9rh/) . Code for statistical analyses is also available in the same OSF page.
