# Supplementary material for: The English Pseudoword Lexicon Project (EPLeP): A resource for investigating pseudoword processing
Source: Behav Res Methods. 2026 Sep 22;58(11):297. doi: 10.3758/s13428-026-03151-5 (PMC13597635; doi:10.3758/s13428-026-03151-5)
Supplement: Supplementary file 1 [file 13428_2026_3151_MOESM1_ESM.docx]

# EPLeP: English Pseudoword Lexicon Project -

# A resource for investigating pseudoword processing

Fabio Marson^1^°, Rolando Bonandrini^1,2^°, Iva Šaban^1^°, Simona Amenta^1^, Marco Marelli^1,2^*

^1^ Department of Psychology, University of Milano-Bicocca, Milano, Italy

^2^ NeuroMI, Milan Center for Neuroscience, Milano, Italy

° These authors equally contributed to this work; * Corresponding author

# Supplementary Materials

## Subsampling of the stimuli

Nonwords and simple pseudowords were subsampled in order to reflect the distribution within the full set of length and OLD20 within their category, while complex pseudowords were sampled in order to reflect the distribution within the full set of length, and OLD20, and family size of the stems.

### Word stimuli

The intermediate stage contained sets of 9865 morphologically simple words, 3119 morphologically complex words and 2049 stems. Each of them has been reduced using the identical subsampling strategy which aimed to maintain the same features of the original set, in terms of orthographic length, family size, and OLD20 (according to both US and UK corpora). That is, for each of these variables, we segmented the total distribution of scores in 50 bins and extracted the absolute number of items in each bin. We then generated a subsample of the data of the desired dimension, computed the distribution for each feature of interest and tested the correlation between the distribution of each variable in the subsample vis-à-vis the full pool. Random sampling was automatically repeated until the correlation between the number of items belonging to each bin of the original distribution and the ones in the subsampled distribution reached at least r > .95 for all the variables of interest. As additional criteria, we checked that the comparisons between the full pool and the subsample yielded p > .05 in both a Kolmogorov-Smirnov and an unpaired two-samples Wilcoxon test.

This subsampling strategy resulted in extracting 200 free-standing stems (e.g., “snow” from “snowy”, but not “alter” from “altercate”) reported in the ukWac, in the Subtlex, in the Morpholex “0-1-0” (0 prefixes, 1 stem, 0 suffixes), and in the Wikipedia-based corpora. They served to generate complex words and complex pseudowords and were also included as standalone simple words.

Using the same subsampling strategy described above, we reduced the set of morphologically simple words to 2500 items. However, after exclusion of unwanted inflected forms and socially unacceptable taboo words (N=86; as identified in the US subset of the multilingual resource by Sulpizio et al., 2024), the least frequent words (N=250) and practice items (N=3), we obtained a final list of 2161 morphologically simple words.

Finally, the set of morphologically complex words underwent the same subsampling strategy, resulting in 2485 items. This number was further reduced after exclusion on the least frequent words (N=250) and practice items (N=3), accounting for a total of 2232 morphologically complex words.

### Simple pseudowords

As described in the manuscript, a starting pool of 2500 simple words was fed into Wuggy in order to obtain 20 associated pseudowords per word. Note that Wuggy was not always able to generate 20 requested associated pseudowords per each simple word (especially for short ones like “eye”).

Prior to the subsampling procedure, we removed repeated items, items with an inflectional suffix attached to an existing word (e.g., “rockest” is a pseudoword but it could be parsed in rock + est, resembling a complex form), and novel compounds (e.g., “pupcar” is a pseudoword which could be parsed in pup + car, both existing English words, see Auch et al., 2023). Since Wuggy could not generate the equal number of pseudowords for each word, in the following subsampling procedure, we extracted a subsample of simple pseudowords to match the desired set size of 4500 stimuli using the same subsampling strategy described above.

We note that we conducted two rounds of subsampling. First, we performed a semi-randomized filtering based on the amount of pseudowords generated from each word: we kept all non-lexical items paired with words that generated only 1 or 2 pseudowords; for words that generated more than 2 pseudowords, we kept 2 pseudowords in 30% of the cases, and 3 in the remaining 70%. We then ended up with 4015 simple pseudowords which was below our initially set target number of 4500 simple pseudowords.

To compensate for this lack of simple pseudowords, we decided to run another subsampling from the 19694 eligible pseudowords. In this round, we excluded repetitions, inflectional suffix, and compound stimuli before the subsampling of the final list of simple pseudowords. Differently from the previous tentative subsampling, in this case we extracted 2 pseudowords generated from each word in 18% cases and 3 pseudowords in 82% of the cases). Thus, we finally obtained a list of 4524 simple pseudowords that matched our requirements.

### Nonwords

The OLD20 was computed based on US spelling, to mirror the spelling used in the MorphoLex corpus. For each length level, we generated 2500 nonwords (2500 * 7=17500), based on the token frequency of each letter in the Subtlex-US corpus. We discarded repeated items and attested words. Finally, a subset of nonwords for each length level was extracted so that the distribution of the final list of nonwords in each length level was representative of the distribution of the whole pool of stimuli generated in all the other categories. To ensure that nonwords were lexically dissimilar from the other categories of pseudowords, we finally ran a set of two-samples Wilcoxon tests to check that both OLD20 US and OLD20 UK were significantly greater (i.e., resulting in less word-like stimuli) for the nonwords list in comparison with all other lists (all p values < 0.001).

### Final item set

The distributions of the final item set for each category of stimuli (simple pseudowords, complex pseudowords, nonwords, simple words, complex words) for length and OLD20 are visualized in Figure 1S and Figure 2S, respectively.

**Figure 1S**

*Distribution of the length (in letters) for each category of generated stimuli*

**Figure 2S**

*Comparison of OLD20 values for each category of stimuli obtained from OLD20-US (left panel) and OLD20-UK (right panel)*

*Note*. #SW=simple words; #CW=complex words; #SPW=simple pseudowords; #CPW=complex pseudowords; #NW=nonwords

## Construction of experimental lists

Experimental lists were constructed by assigning ranks from 1 to 225 to stimuli in decreasing order of frequency (Zipf for simple and complex words) or OLD20 (for unattested stimuli lists), until completion of each stimulus list. Then, each code was used to assign the stimulus to the corresponding experimental lists (e.g., the stimulus “1” will go in list 1, stimulus “2” in list 2, and so on until completion). In this way we managed to produce experimental lists of words balanced for frequency (all p > .99) and lists of non-lexical items balanced for OLD20 (all p > .99).

### Catch trials

For what concerns words, we identified the 10 most frequent words (in terms of Zipf US) from each experimental list as catch trials as they are supposed to be the easiest to be classified as words. For what concerns unattested stimuli, we identified the 10 items with the highest OLD20 US values from each experimental list as catch trials since these are supposed to be the least word-like stimuli, thus easiest to reject. All the catch trials resulted to be balanced across experimental lists for what concerns frequency (all p > .93) and OLD20 (all p > .97).

### Selection of practice trials

Practice trials were selected from one lexical category (i.e., words) and three non-lexical categories (i.e., simple pseudowords, complex pseudowords, nonwords). Their orthographic length was representative of the whole set. We divided each set of non-lexical stimuli in 6 quantiles and extracted one random item from each quantile. To obtain a set of 6 practice trials for words, we used the same strategy to extract three items from the simple and three items from the complex word sets. Stimuli for practice trials were sampled from four categories of stimuli and are listed in Table 1S.

**Table 1S**

*List of practice trials extracted from each category of stimuli*

| Category | Stimuli |
| --- | --- |
| Simple Pseudowords | etase, trorn, woolon, ahucan, coluntym, mandafad |
| Complex Pseudowords | lowify, medally, housion, variness, chirpence, basketery |
| Nonwords | nrtlg, hsmxu, ypyegi, iyawohh, wslhrhmi, xueeewggt |
| Words | prey, bullet, license, useful, roughly, idleness |

*Note*. For the Words category, the first three were extracted from the simple words list, the latter three from the complex words list

## Reference list

Auch, L., Pérez Cruz, K., Gagné, C. L., & Spalding, T. L. (2023). LaDEP: A large database of English pseudo-compounds. *Behavior Research Methods*, *56*(3), 2606–2622. https://doi.org/10.3758/s13428-023-02170-w

Sulpizio, S., Günther, F., Badan, L., Basclain, B., Brysbaert, M., Chan, Y. L., Ciaccio, L. A., Dudschig, C., Duñabeitia, J. A., Fasoli, F., Ferrand, L., Filipović Đurđević, D., Guerra, E., Hollis, G., Job, R., Jornkokgoud, K., Kahraman, H., Kgolo-Lotshwao, N., Kinoshita, S., … Marelli, M. (2024). Taboo language across the globe: A multi-lab study. *Behavior Research Methods*, *56*(4), 3794–3813. https://doi.org/10.3758/s13428-024-02376-6
